# A novel (targeted) kinesio taping application on chronic low back pain: Randomized clinical trial

**DOI:** 10.1371/journal.pone.0250686

**Published:** 2021-05-13

**Authors:** María Lourdes Peñalver-Barrios, Juan Francisco Lisón, Javier Ballester-Salvador, Julia Schmitt, Aida Ezzedinne-Angulo, María Dolores Arguisuelas, Julio Doménech

**Affiliations:** 1 Department of Physical Medicine and Rehabilitation, Hospital Arnau de Vilanova, Valencia, Spain; 2 Department of Medicine and Surgery, Universidad Cardenal Herrera CEU, CEU Universities, Valencia, Spain; 3 Department of Biomedical Sciences, Universidad Cardenal Herrera CEU, CEU Universities, Valencia, Spain; 4 CIBER Fisiopatología Obesidad y Nutrición (CB06/03), Instituto Carlos III, Madrid, Spain; 5 Department of Nursing and Physiotherapy, Universidad Cardenal Herrera CEU, CEU Universities, Valencia, Spain; 6 Department of Orthopaedic Surgery, Hospital Arnau de Vilanova, Valencia, Spain; California State University San Marcos, UNITED STATES

## Abstract

The aim of the present clinical trial is to evaluate the efficacy of kinesio taping on patients with chronic low back pain, when the exploration identifies skin/fascia mobilization as a factor that could modify the treatment effect. This study is a randomized controlled trial with intention-to-treat analysis. Sixty-two participants with chronic low back pain were therefore recruited from a tertiary referral hospital. Targeted kinesio taping, according to skin/fascia mobility exploration, was applied in the experimental group **(17 female/13 male; 49.47 ± 11.15 years)** once a week for four sessions. The control group **(17 female/14 male; 48.87 ± 9.09 years)** underwent a placebo taping application. At post-treatment time there was a statistically significant **reduction** both in disability (Roland-Morris Disability Questionnaire) and pain (Numeric Pain Rating Scale) in **the experimental group (disability: −2.88, 95% confidence interval [CI] −4.56 to −1.21, P < .001; pain: −1.58, 95% CI −2.67 to −0.54 P = .001) and the control group (disability: −1.82, 95% CI −3.46 to −0.17 P = .025; pain: −1.30, 95% CI −2.32 to −0.28 P = .008)**. However, at six months, these changes only remained significant in the experimental group (disability: −2.95, 95% CI −4.72 to −1.18, P < .001; pain: −1.06, 95% CI −2.07 to −0.04, P < .05). As a conclusion, the application of targeted kinesio taping produced a significant reduction in pain and disability, at **4 weeks and at** 6 moths **follow-up**, **although there were no differences between groups at any measurement time point**.

## Introduction

Non-specific low back pain is, nowadays, one of the main health problems in developed populations, not only for its high prevalence (ranging from 22% to 65%) [1] but also for its high chronicity percentage and the involved disability [2,3]. Moreover, the absenteeism and loss of productivity of patients suffering from non-specific low back pain leads in an important economic impact [4].

Determining the effectiveness of different approaches used for treating chronic low bak pain (CLBP) is a priority for healthcare systems [3,5]. Physical therapy is one of the fundamental pillars in treating CLBP [6,7]. The most recent published work regarding non-invasive therapies in low back pain includes data on the use of Kinesio Taping (KT) [7].

Evidence from randomized clinical trials (RCTs) focused on assessing the efficacy of KT in CLBP has a wide range of results. **The duration of the KT intervention, ranging from 24 hours to 4 weeks, indicates a different characteristic between the studies.** Some of them have not found additional benefit of KT when combining with other physical therapies [8–11], however, Koroglu et al. stated that KT is an effective method which increases the effectiveness of the treatment in a short period when applied in addition to exercise and electrotherapy methods [12]. Others studies have found improvements in pain and disability using KT versus placebo in the short term, although these effects were very small to be clinically worthwhile [13,14]. Contrarily to this, some authors have reported that KT does not seem superior than placebo taping, immediately postreatment, in terms of reducing pain or disability [15–18].

A recent meta-analysis **about chronic low back pain**, concluded that although KT was not superior to placebo taping in terms of alleviating pain intensity, it did significantly improve disability [19]. Standard taping techniques were used in all these trials for all the experimental-group patients, and in most cases this treatment consisted of a ‘muscular technique’ applied to the lumbar paravertebral musculature [8–10,14–17], a ‘star-shaped space technique’ applied in the area where the pain was most acute [13,17,20], a combination of both these techniques [11], or a ‘transversal space technique’ at several lumbar levels [12]. None of these studies adapted the application of KT to the specific clinical characteristics of the patient, yet some published studies about CLBP state that clinical results can be improved by targeting treatments to different patient subgroups [21,22].

Indeed, many clinicians recognize that they base their therapeutic decisions on the patterns of signs and symptoms seen in each patient without requiring a prerequisite diagnosis of an anatomical injury [23]. This has led to the emergence of the concept of ‘treatment effect modifiers´ (TEMs) which describes characteristics that identify patient subgroups that respond differently to specific interventions [24].

On the other hand, lumbar fascia has been suggested to be involved in CLBP [25]. **The fascia is a continue structure throughout the human body which allows numerous sliding directions. It has been suggested that mechanical forces applied at the macroscale produce changes in biochemistry and gene expression within individual living cells. This phenomenon called mechanotransduction is the basis to explain how physical manipulation may influence tissue physiology [26–28]**. **Given that** fascia tissue displacement is **diminished in LBP** patients [**29],** manual skin/fascia mobilization in different directions would be able to modify a patient’s signs or symptoms, considering this exploratory maneuver a TEM. **In the present study we developed a novel kinesiotaping application considering the previous skin/fascia mobilization, an issue which has not yet been arisen.** Therefore, this study aimed to evaluate the short and medium-term efficacy of treatment with KT in patients with CLBP in terms of disability, pain, and quality-of-life when their initial exploration indicated that skin/fascia mobilization was a possible TEM.

## Materials and methods

### Study design

**Fig 1:**
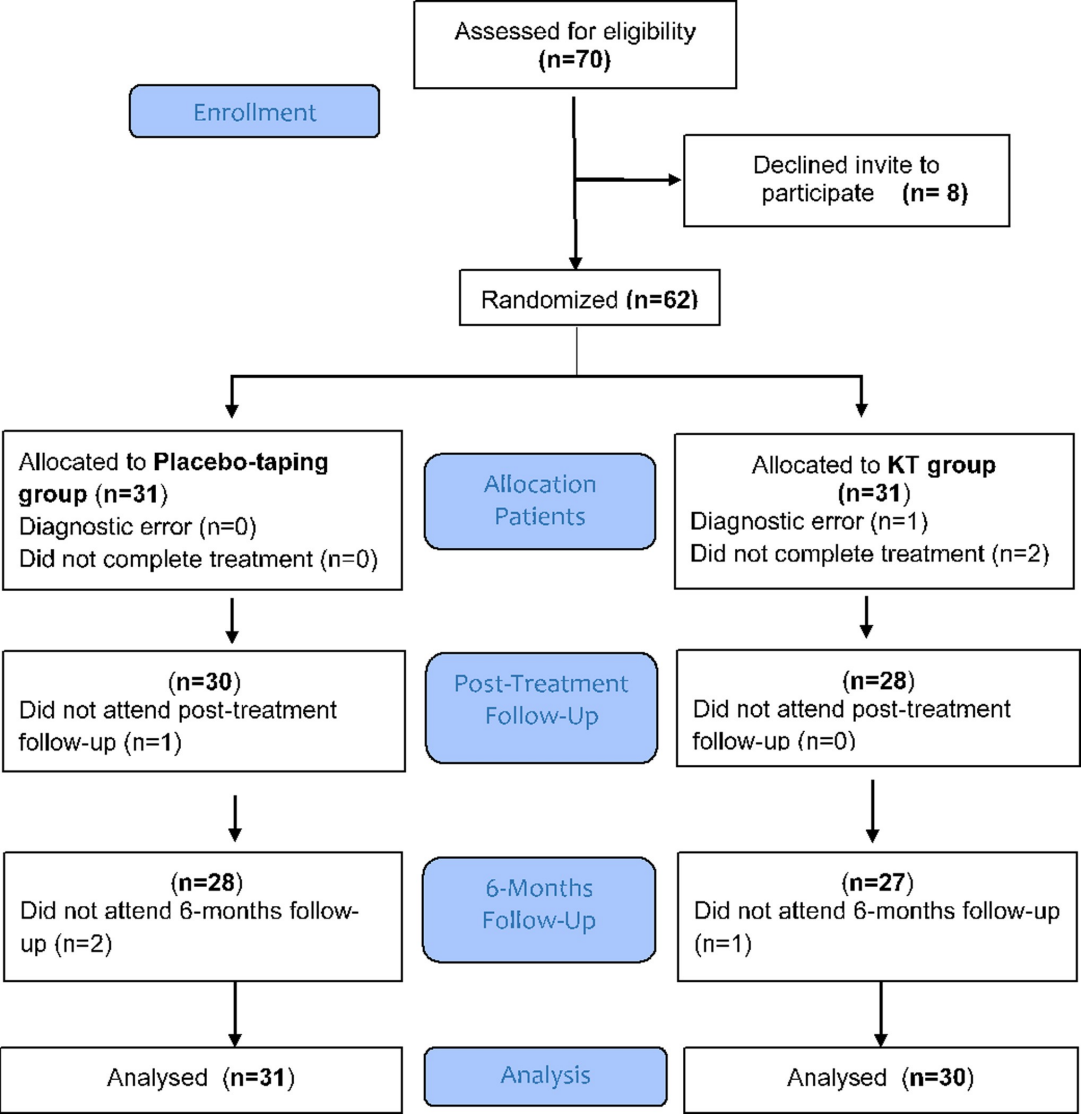
Flow diagram of the study.

This was a randomized, double-blinded, parallel clinical trial with two treatment arms in which 1:1 allocation was used. The participants were recruited from December 2015 to November 2016 at the outpatient service of a Physical Medicine and Rehabilitation Service and they all signed their informed consent to participation. Fig 1 shows the progression of the participants through the trial. The project was approved by the Research Ethics Committee at Hospital Arnau de Vilanova in November 2012 and followed the ethical guidelines set out in the Declaration of Helsinki. The evaluation was carried out in three stages: pre-intervention, post-intervention, and 6 months after the intervention.

An investigator from the Physical Medicine and Rehabilitation Service was responsible for enrolling the participants, and a statistician outside this research team generated a random number list using random number allocation software [30]. The randomized sequence was concealed via cards stored in sealed, numbered, opaque envelopes which, in order to preserve allocation concealment, were opened only after the patient confirmed their decision to participate in the trial. During the follow-up, the study participants and the investigator assessing the outcomes remained blinded to the patient group assignments. The trial was registered at ClinicalTrials.gov, NCT02604290.

### Participants

The following inclusion criteria were applied: 1) age between 18 and 65 years; 2) non-specific CLBP present for at least 6 months; 3) a disability score equal to or greater than 4 on the *Roland-Morris Disability Questionnaire* (RMDQ); 4) improvement of pain with skin/fascia mobilization during the initial exploration and therefore considered a TEM. The exclusion criteria were: 1) previous experience with KT treatments; 2) neuropathic pain component (e.g., radiculopathy or lumbar canal stenosis); 3) pain of a specific origin (e.g., vertebral fracture, neoplasm, inflammatory spondyloarthritis, or spondylodiscitis); 4) prior lumbar spinal surgery; 5) mental disability, severe mental illness, abuse or dependence on substances, illiteracy; 6) allergy or dermal lesions that could prevent the application of tapes.

### Intervention

Each participant received a physical examination at the beginning of the study by a physician. The clinical examination included: 1) pain detection with active lumbar mobility; 2) palpation in the prone position while applying Maigne segmental exploratory maneuvers [31]; 3) active lumbar mobility while the clinician manually mobilizes soft tissues by displacing the skin, in different directions, in painful segmental levels. The latter maneuver was considered a TEM if it caused the patient’s pain to decrease or subside.

Before being randomized into the KT or placebo-taping group, the basic rules of postural control and tonic lumbar stabilization were explained to all the patients. In addition, they were all also given a photocopy of a basic set of quadruped exercises and were instructed to perform them daily.

**Fig 2:**
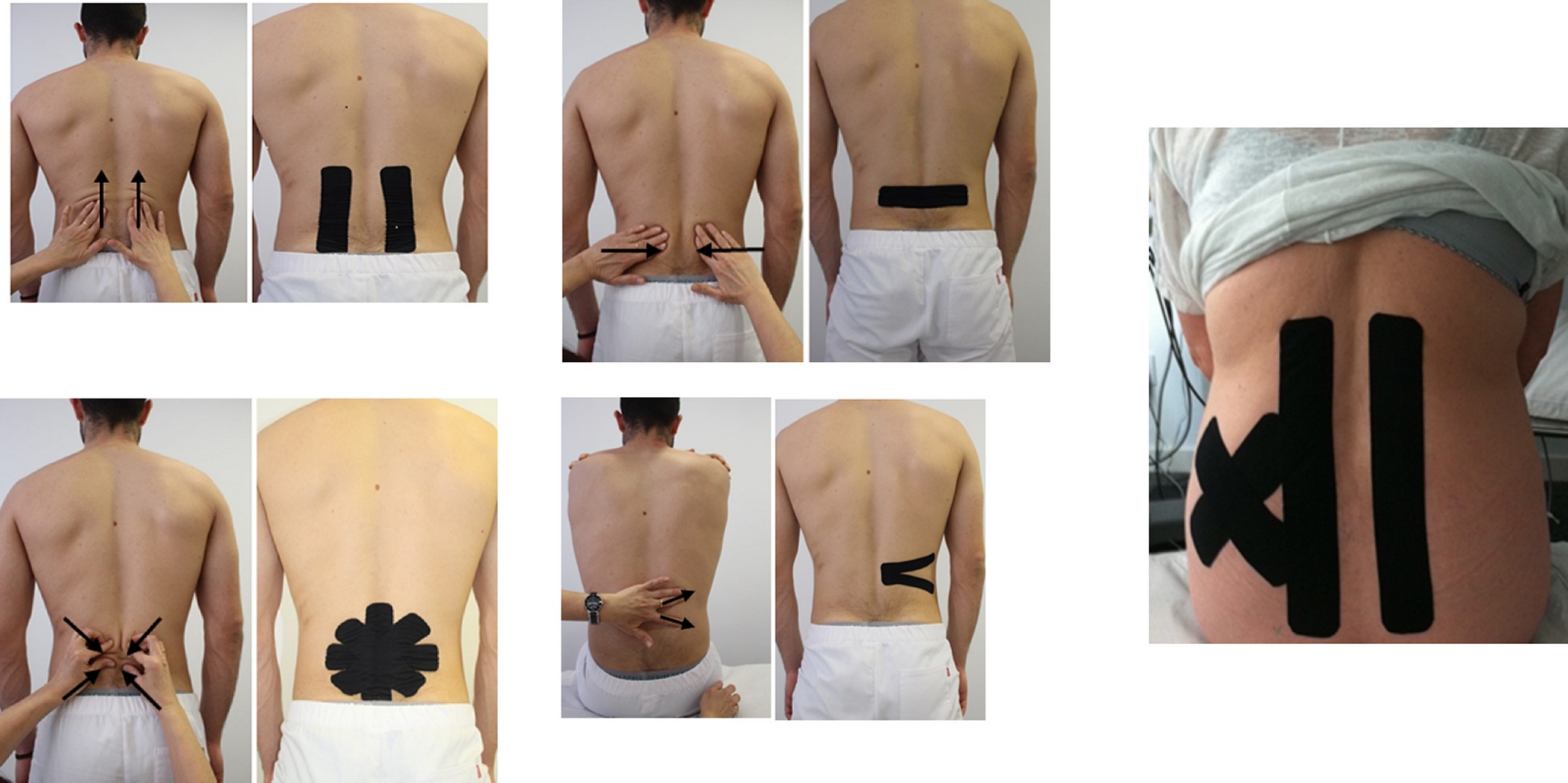
Targeted therapy with kinesio taping: The arrows indicate the direction of the skin/fascia mobilization. A) Muscle technique, with application starting from the upper region and extending down over the paravertebral muscles. B) Transversal space technique with application at the segmental level. C) Star space technique with application at the segmental level. D) Fascia technique with application over the muscular band of the quadratus lumborum. E) Muscle technique over the paravertebral muscles and X space technique over the iliac crest.

**Fig 3:**
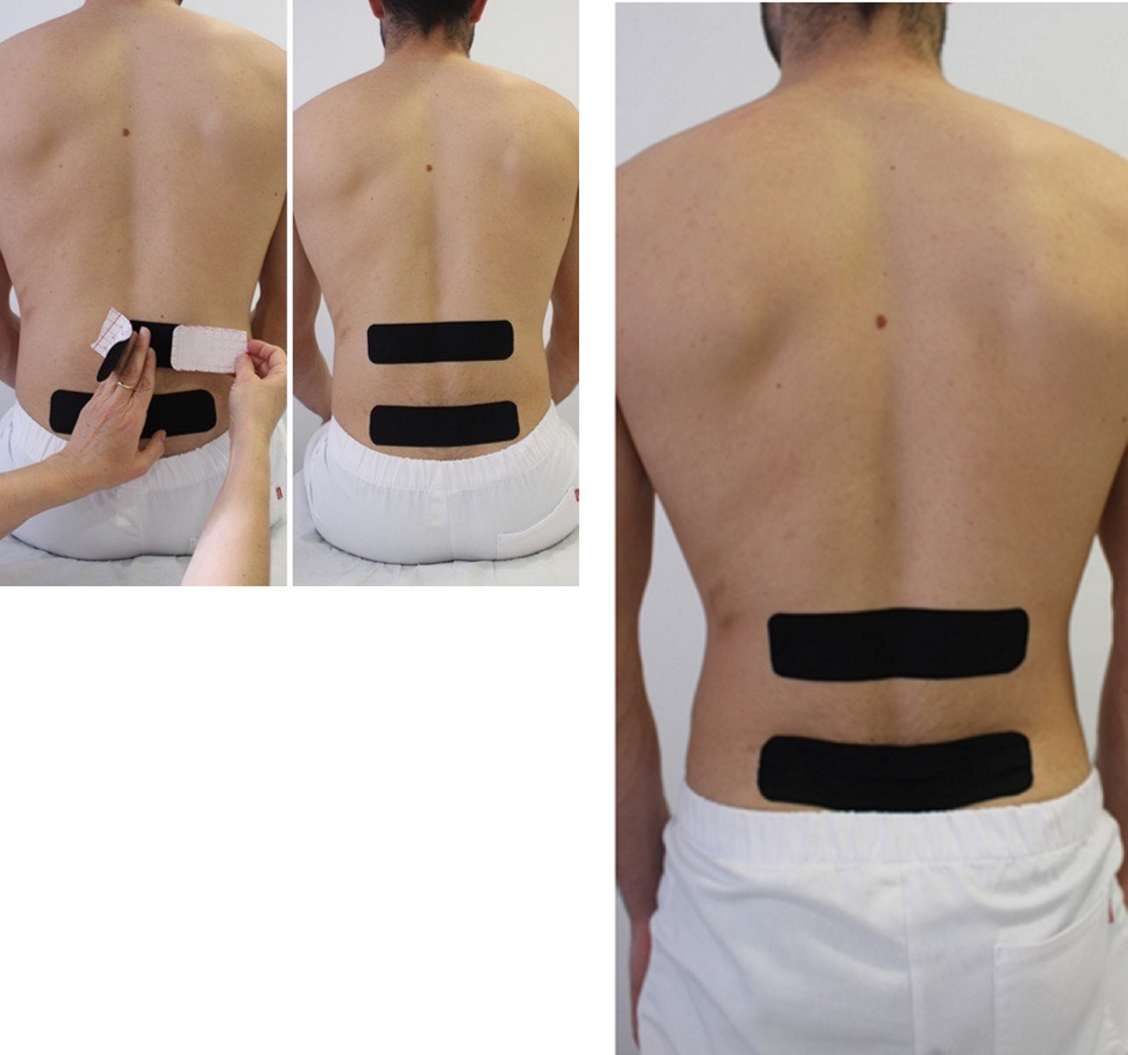
Placebo-taping application. A-B) Two transversal tape strips were placed at two segmental levels that were not painful upon palpation, with the patient always in a neutral body position and without applying tension at any point along the tape. C) When standing, no ripples were observed in the bandage.

Targeted therapy was applied in each session in the KT group, according to the following guidelines: 1) if the pain, during active lumbar mobility, decreased when longitudinally mobilizing the skin/fascia over the paravertebral musculature fibers, the “muscle technique” was applied (Fig 2A) (The tape is applied longitudinally to the paravertebral musculature. With the patient sitting in a neutral position, the base of the tape is located at the end towards which the mobilization has been directed in the clinical examination. Once the base has been applied without tension, the patient is asked to perform the maximum lumbar flexion he can tolerate and in this posture the rest of the tape is also applied with 0% tension); 2) If the pain, during active lumbar mobility, decreased when mobilizing the skin/fascia towards a certain point, the “space technique” was applied (The tape is applied centered on the painful area indicated by the examination. The patient should be placed in the maximum possible lumbar flexion, since the sitting position. A tension of 50 to 75% is used in the center of the tape and 0% of tension at the ends. Usually one or more parallel horizontal strips are used (Fig 2B), but sometimes several strips can be used forming an X or a star, depending on the exploration (Fig 2C); 3) If the pain, during active lumbar mobility, decreased when transversally mobilizing the skin/fascia over the paravertebral muscle fibers, the “fascia technique” was applied (Fig 2D) (The tape is applied on the taut band in a transverse direction to its fibers. The patient should be placed in the maximum possible lumbar flexion, since the sitting position. The tape is cut in half, except the base. The base is placed without tension and the tails are pulled with short shakes in the desired direction that the exploration has previously indicated); 4) depending on the results of the clinical examination, several techniques were sometimes combined in the same patient (Fig 2E).

In the placebo group, two strips of the same tape were applied in a neutral body sitting position and at non-painful segmental levels, without applying tension at any point along the tape (Fig 3A–3C).

The same doctor carried out all the interventions. Participants in both groups were advised to maintain the tape for seven days [13], if possible, until the following session. All the subjects received a treatment once a week for four weeks. The participants maintained their usual physical activity level and pharmaceutical treatment for LBP during the duration of the study.

### Outcome measures

The primary outcome of the study was changes in the level of disability. This variable was assessed by means of the Validated Spanish version of the Roland-Morris Disability Questionnaire (RMDQ) [32] with scores ranging from 0 (no disability) to 24 (severe disability).

The secondary outcomes of the study were pain and quality-of-life. Pain perception was measured by means of the *Numeric Pain Rating Scale* (NPRS), in which 0 represents ‘no pain’ and 10 ‘the maximum pain imaginable’; *EuroQol five-dimensions questionnaire five-level version* (EuroQol 5D-5L) is a self-administered questionnaire which includes a *EQ Visual Analog Scale* (EQ-VAS) scored from 0, ‘the worst health you can imagine’ to 100, ‘the best health you can imagine’ and the *EQ-5D-5L Index Value* (EQ-INDEX) which, in the Spanish population, ranges from −0.654, ‘the worst possible quality of life’ and 1, ‘complete well-being’ [33].

### Data analysis

The desired sample size was calculated (G-Power, Version 3.1.9.2) taking disability as the main variable, with a minimum clinically relevant difference of 3 points in the RMDQ score [34]. Considering a standard deviation of 3.2, an alpha value of 0.05 and a statistical power of 90% for comparison of two-tailed means, as well as possible losses of 20%, the required study sample size was established as 62 patients.

The statistical analysis was performed based on an intention-to-treat approach. Normality of the variables was confirmed using the Shapiro-Wilk test. Two-way mixed ANCOVA tests were used to compare the effects of the intervention on the outcome metrics (disability, pain, EQ-VAS, and EQ-INDEX) between the groups, using time (pre-treatment, post-treatment, and 6-month follow-up) as the within-group factor, and the intervention type (KT or placebo taping) as the between-group factor. The analysis was adjusted for pre-intervention (baseline) data. To determine the independent relationship between ´pre-treatment´ disability and pain scores and the ´6-month minus pre-treatment´ disability or pain scores, the Pearson correlation coefficient was also calculated. Chi-squared tests were used to determine if there were any differences between the groups in terms of adherence to the prescribed quadruped exercises. The statistical analyses were performed using SPSS Statistics software, version 17.0 (SPSS Inc., Chicago, IL^©^), considering a probability of *p* < .05 as a statistically significant result.

## Results

A total of 70 participants were consecutively recruited to this study. Eight subjects were excluded for the reasons presented in the flow diagram (Fig 1). The remaining 62 participants were assigned randomly into two groups. After randomization, a diagnostic error was detected in one participant from the intervention group. One participant from the placebo group did not attend post-treatment assessment and three participants did not attend 6 months follow-up; experimental group (n = 1), and placebo group (n = 2). All participants in each group were included in the final analysis, with the exception of the diagnostic error.

Table 1 shows the baseline demographic and clinical characteristics of the study participants; no differences were observed between the groups at baseline.

**Table 1:** Demographic and baseline characteristics of the sample.

	Placebo taping (n = 31)	Kinesio taping (n = 30)
**Age (years)**	48.87 (9.09)	49.47 (11.15)
**Sex (female)**	17 (54.8%)	17 (56.6%)
**Weight (kg)**	76.21 (16.13)	80.95 (17.19)
**Height (m)**	1.67 (0.08)	1.68 (0.10)
**BMI (kg/m**^**2**^**)**	25.75 (5.87)	27.59 (4.34)
**Evolution time (months)**	36 (96)	36 (49)
**Employment status**		
Actively employed	12 (38.71%)	14 (46.67%)
Incapacitated (temporary)	0 (0%)	3 (10.00%)
Incapacitated (permanent)	1 (3.22%)	2 (6.67%)
Unemployed (health reasons)	2 (6.45%)	0 (0%)
Unemployed (other)	9 (29.03%)	6 (20.00%)
Student	0 (0%)	1 (3.33%)
Homemaker	6 (19.35%)	3 (10.00%)
Retired	1 (3.22%)	1 (3.33%)
**Charlson Comorbidity Index**	1.50 (1.54)	1.28 (1.66)
**Pre-treatment values**		
RMDQ	8.39 (4.28)	10.20 (4.05)
NPRS	5.93 (1.73)	6.80 (2.26)
EQ-index	0.59 (0.21)	0.60 (1.89)
EQ-VAS	57.50 (18.23)	59.67 (18.61)

Values presented as the mean (SD) or n (%).

### Effects of the intervention

**Fig 4:**
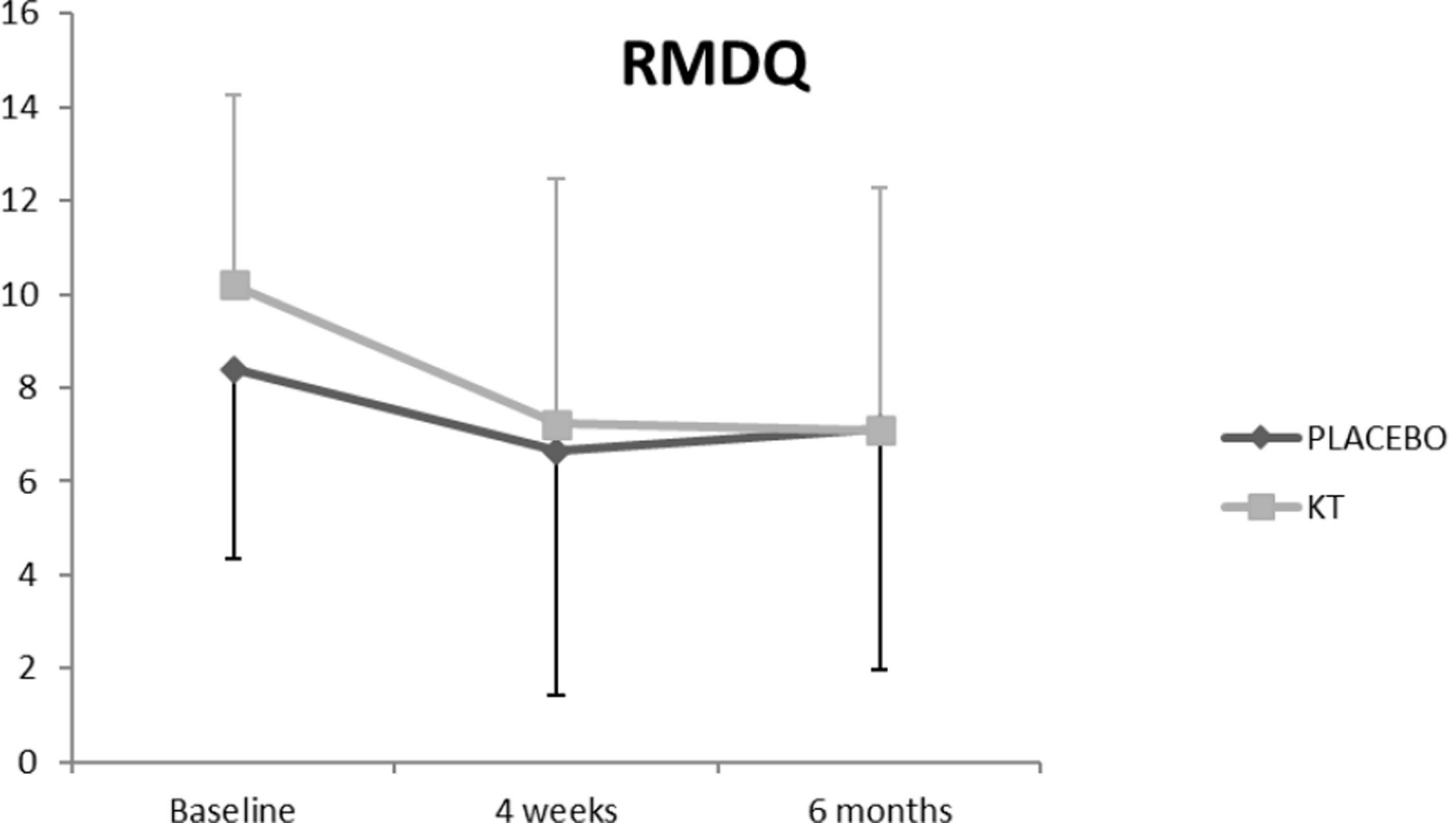
Effects of the intervention on RMDQ.

**Fig 5:**
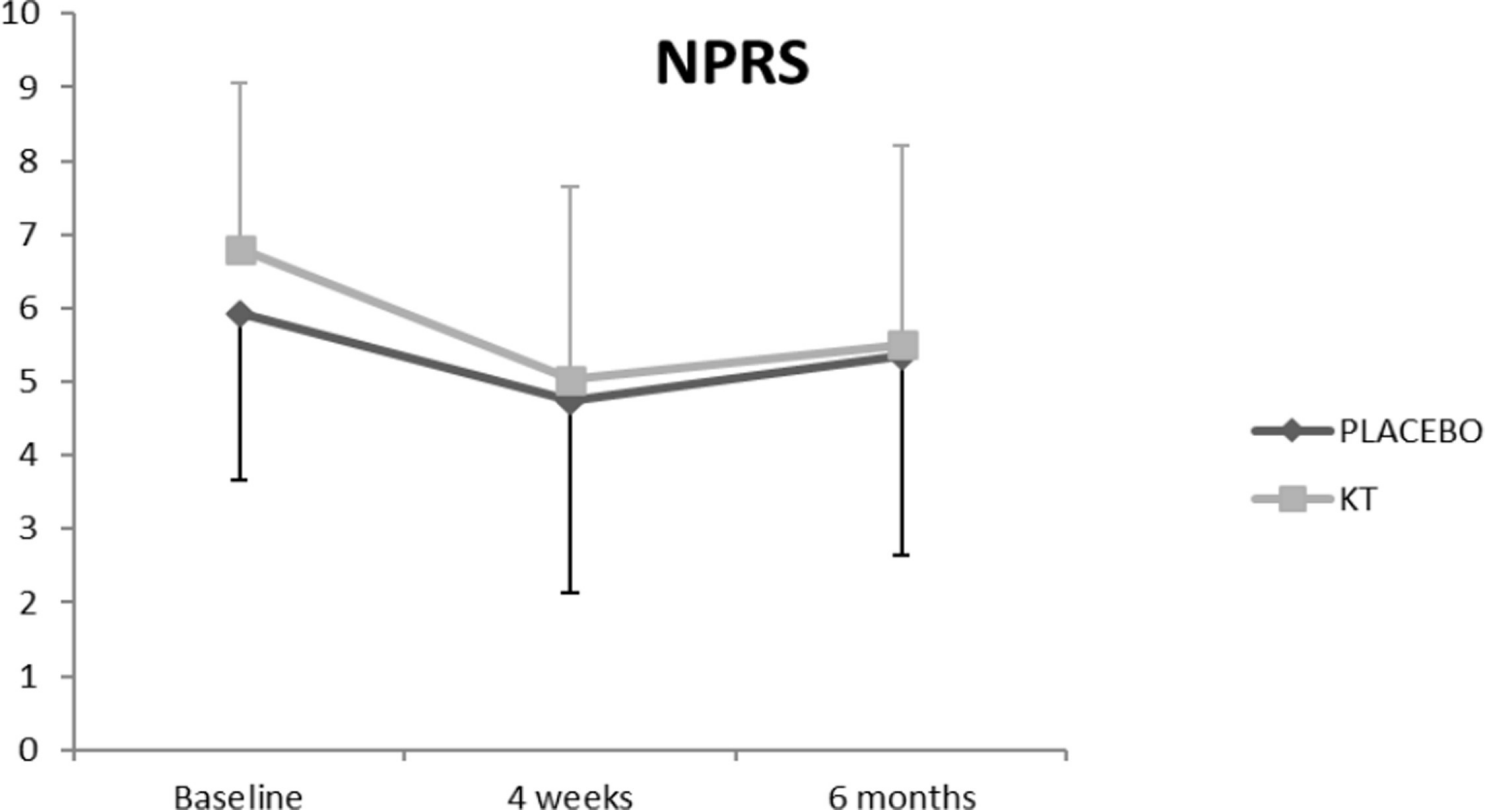
Effects of the intervention on NPRS.

**Fig 6:**
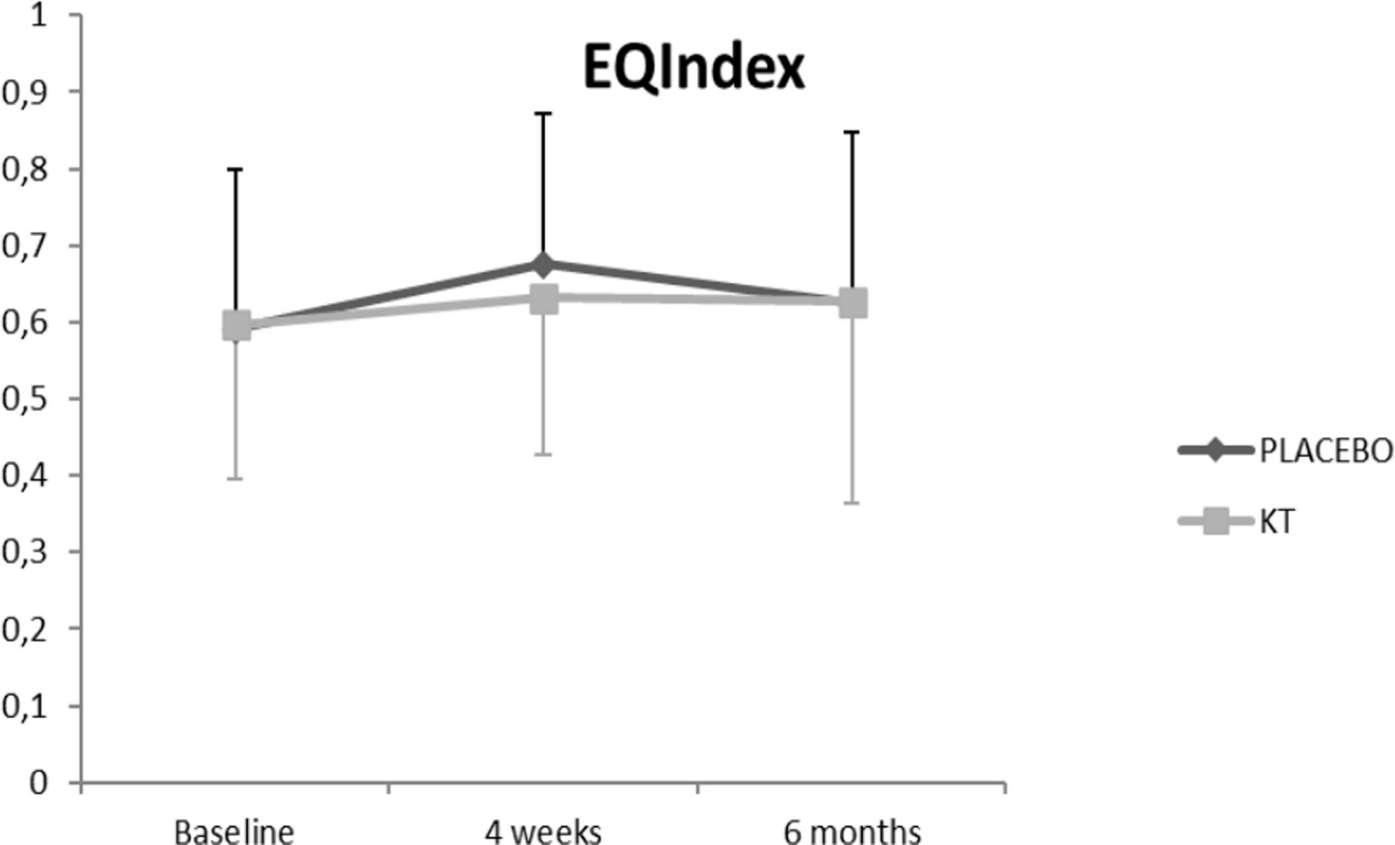
Effects of the intervention on EQ Index.

**Fig 7:**
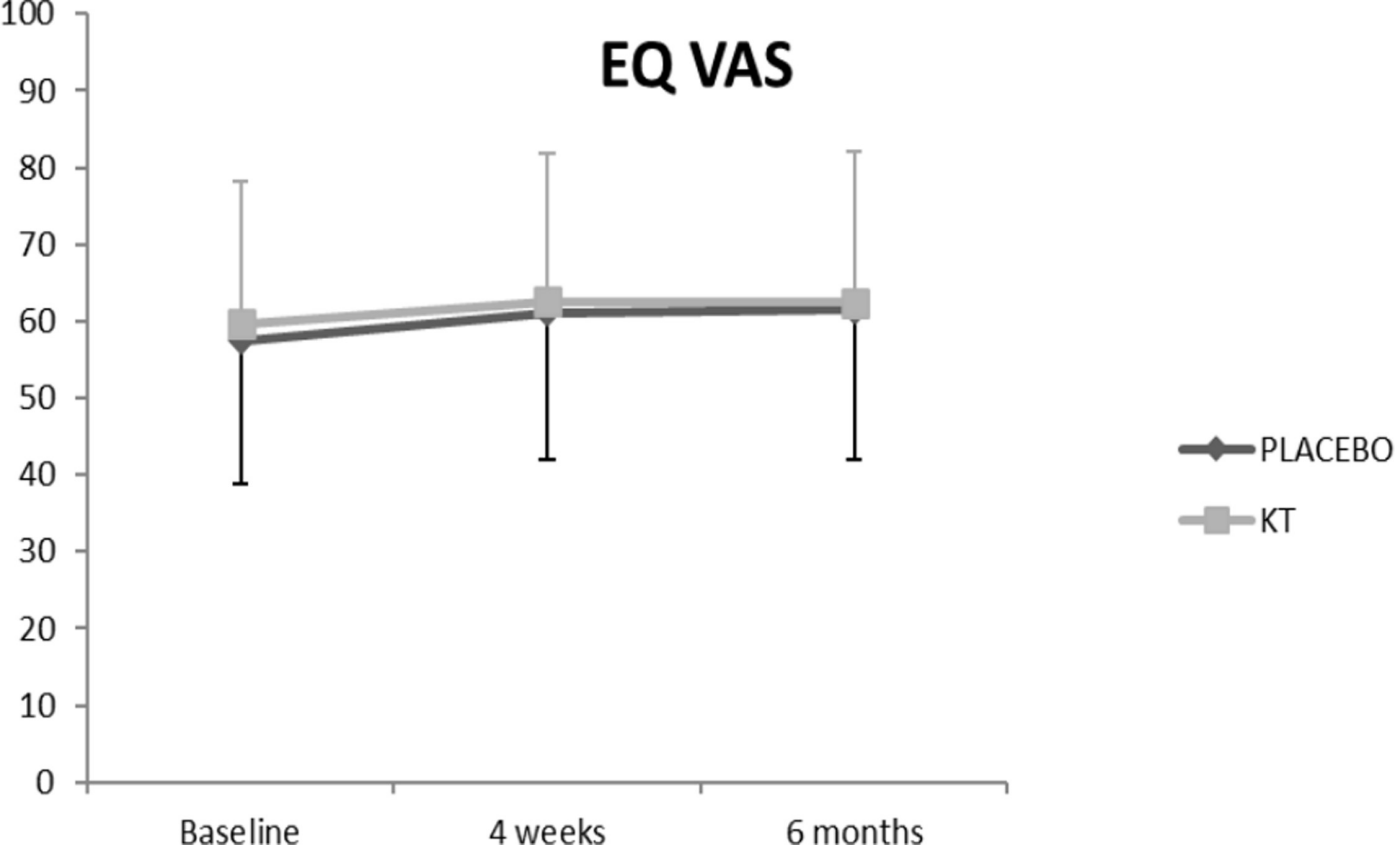
Effects of the intervention on EQ VAS.

The ANCOVA analysis of the main effects showed a significant difference between the time series **(RMDQ, p < 0.001; NPRS, p < 0.001; EQ-index, p = 0.04)**. **Figs 4–7 show the changes in these variables across time and within condition.** The intra-group post-treatment analysis showed a statistically significant difference in terms of the level of disability and pain reduction in both groups and the quality-of-life (evaluated with the EQ-Index) in the placebo group. The intra-group analysis at the 6-month follow up showed a statistically significant difference in terms of level of disability and pain reduction only in the KT group. The inter-group analysis showed no differences in any of the comparisons (Table 2).

**Table 2:** Results of the variables (pre-treatment, post-treatment, and at 6 months) and difference within groups (post-treatment and at 6 months).

Variables	Descriptive data				Difference within groups			
	Group	Pre-treatment	Post-treatment	6 months	Post-treatment minus pre-treatment	Cohen’s d	6 months minus pre-treatment	Cohen’s d
**RMDQ**	**KT**	10.20 (4.05)	7.23 (5.23)	7.10 (5.17)	−2.88** (−4.56 to−1.21)	0,635	−2.95** (−4.72 to −1.18)	0,668
	**Placebo taping**	8.39 (4.28)	6.64 (5.50)	7.13 (5.24)	−1.82* (−3.46 to−0.17)	0,355	−1.40 (−3.13 to 0.33)	0,263
**NPRS**	**KT**	6.80 (2.26)	5.03 (2.62)	5.50 (2.71)	−1.58** (−2.67 to−0.54)	0,723	−1.06* (−2.07 to−0.04)	0,521
	**Placebo taping**	5.93 (1.73)	4.74 (2.53)	5.35 (2.24)	−1.30* (−2.32 to−0.28)	0,549	−0.58 (−1.58 to 0.41)	0,29
**EQ-index**	**KT**	0.596 (1.889)	0.632 (0.204)	0.627 (0.263)	0.04 (−0.04 to 0.11)	0,027	0.03 (−0.06 to 0.13)	0,023
	**Placebo taping**	0.591 (0.209)	0.676 (0.196)	0.625 (0.223)	0.08* (0.01 to 0.15)	0,42	0.03 (−0.06 to 0.12)	0,157
**EQ-VAS**	**KT**	59.67 (18.61)	62.63 (19.31)	62.50 (19.64)	3.78 (−4.74 to 12.31)	0,156	3.50 (−5 to 12)	0,148
	**Placebo taping**	57.50 (18.23)	61.17 (19.24)	61.50 (20.35)	2.85 (−5.67 to 11.37)	0,196	3.33 (−5.16 to 11.83)	0,207

Values presented as the *mean (SD)* and *difference between the means (95% CI);*

*p ≤ .05,

**p ≤ .001.

The Chi-square test showed no significant differences between the groups in terms of the proportion of patients who said they had done the prescribed exercises and those who admitted not having completed them (*p* = 0.817). The incidence of adverse effects **over all the sample** was low, and these included itching **(4.92%),** dermatitis **(6.55%),** or excessive adhesion of the acrylic adhesive **(1.64%).** The Chi-square test did not show any significant differences between either group in the incidence of these effects. The Pearson correlation results indicated that a higher level of initial disability did not significantly correspond to a greater reduction in disability at the 6-month follow-up. However, higher initial levels of pain intensity did correspond to a greater reduction in pain at the 6-month follow up.

## Discussion

To the best of our knowledge, this is the first prospective, randomized, double-blind controlled trial to explore the efficacy of KT in patients with CLBP using the mobilization of skin/fascia during their initial exploration, as a possible TEM.

A statistically significant reduction in the level of disability and pain intensity was identified, in both groups, **at 4 weeks versus baseline**. These results are in agreement with other studies published so far [14,17,18] which also investigated the effects of KT in patients with LBP. **Pain and disability scores of our participants were similar than those reported in these studies at baseline, with the exception of Keles et al. [17] who reported a higher (moderate) disability level.** Moreover, these changes only remained statistically significant in the KT group at the 6-month follow-up.

Although the reduction in the disability score in the KT group, at 6- month follow-up, was near to three points, that has been set the minimum clinically relevant difference (MCRD) [34], it is not possible to conclude that this improvement is clinically relevant.

Regarding quality-of-life assessment, there was no significant intra-group change at 6 months. Indeed, a study evaluating the sensitivity to change of the outcome measures in patients undergoing spinal surgery concluded that, compared with the pain and disability scales, the SF-36 and EuroQol-5D quality-of-life scales are not sensitive enough to detect clinical changes [35]. Thus, this fact could potentially justify that the reduction in the level of disability and pain observed in the KT group at 6-months follow-up was not accompanied by a significant improvement in the quality-of-life score.

At the 6-month follow-up, the results of this trial did not show any significant differences in, any variable, between both treatment groups. These results concur with those from the only one previous RCT that has collected data from the 6-month follow-up point in patients with non-specific CLBP, after a conventional application of KT [36].

Only the KT group showed a **significant intragroup reduction** in medium-term (at 6 months) in disability and pain scores, however this difference was not significant when compared with the placebo-taping group. The mechanism by which the KT application would cause a long-lasting reduction in disability and pain is not clear. One of the differences between the present study and other studies not reporting medium-term effects of KT, apart from considering skin/fascia mobilization as a TEM, is the duration of the taping application. Our intervention protocol lasted four weeks, while other treatment protocols registered the effect of 7-day KT [13,18], 2- week KT intervention [14] or a maximum of 3-week KT treatment [17]. According to the impact duration of the taping, it would be possible that the results of the present study were maintened for a longer time period than those providing shorter KT interventions. Although the physiological mechanisms involved in the effects of KT extends beyond the scope of this study, the **reduction** in pain and disability observed in the KT group may be due to a neuromuscular input to the central nervous system originating from mechanoreceptors embedded in the fascia. The KT applied according to the exploration of the skin/fascia mobility could lead to a stimulation of these mechanoreceptors which could elicit a neural feedback providing the participants a modified movement pattern which could lead, temporarily, in a better performance of daily activities. The neuroreflex mechanism could also be related to the biological theory of tensegrity/mechanotransduction, according to which physical forces are able to regulate cellular biochemical responses [28].

On the other hand, the clinical improvement seen in the placebo group may be the result of these patients’ natural clinical progression, the regression to the mean phenomenon, co-interventions, researcher and patient biases, or be because of the real placebo effect [37,38].

Prospective cohort studies show that lumbar pain persists in 65% of patients at the one-year follow-up [6], and clinical-course studies have identified different “trajectories of pain” [39], some of them stable in the long term, even up to 7 years [40]. There is a higher prevalence of persistent pain trajectories in primary care patients [39], such as the great majority of patients seen in our Physical Medicine and Rehabilitation Service. Therefore, natural patient evolution was discarded as a cause of the clinical improvement which was seen in the patients included in our RCT.

The correlation between the pre-treatment values and the ‘6-month minus pre-treatment’ difference led us to conclude that, although it is possible that the regression to the mean phenomenon could have influenced the pain results, it is unlikely to explain why a clinical improvement in the level of disability was seen. Moreover, the RCT study design is recognized as the best method for mitigating the regression to the mean phenomenon [41] and this same design allowed us to rigorously control potential selection, action, application, detection, and wear biases in the research reported here.

Far from being inert, real placebos—the use of patient psychosocial contexts [42]—have genuine effects [38] which are mediated by psychological and physiological changes in patients’ pain perception [37]. These include expectation and, in the treatment of pain, activation of the opioid system. Therefore, a placebo component (a non-specific effect) is implicit in the mechanism of action of all treatments used in clinical practice, and these may or may not be associated with a specific component [37]. Furthermore, the strongest placebo effects are seen in physical placebo interventions, outcomes specifically involving the patient, small trials [43], or culturally-innovative interventions, all of which were conditions present in our trial.

Given that, with few exceptions, therapeutic interventions cannot be decontextualized from their psychosocial contexts, several authors have warned that RCT designs with placebo controls may underestimate treatment effects [38]. Patients’ positive expectations about the assigned intervention can improve their eventual results and, even more surprisingly, placebos without deception are still significantly more effective than the absence of treatment, and can significantly increase the efficacy of standard treatment regimens [44]. In fact, the most recent American Medical Association guidelines for the use of placebos proposes that a phrase such as “placebos activate specific brain circuitry that produces relief of symptoms” be used when explaining the details of treatment to patients [45].

Our results showed a reduction in pain and disability at 6 months follow up only in the KT group, although **there were no differences in these outcomes between groups**. Maybe, future studies with larger sample size could confirm differences between groups at medium-term. Moreover, a recent systematic review including sixteen RCT investigating KT effects in patients with CLBP, concluded that there is strong evidence that KT improves pain and disability in this population [46]. According to the tendency of our results and supported by the conclusions of the most recent review we consider KT as a possible tool for clinicians to manage CLBP.

### Study limitations

One limitation of this trial was that it did not include a third untreated group to objectify the extent to which the improvement seen in the placebo group was the result of natural patient evolution, the regression to the mean phenomenon, or to the true placebo effect [38]. Furthermore, the study sample was recruited in a tertiary center and all the patients had long-term CLBP meaning that these results may not be applicable in patients who have experienced lower back pain for shorter periods. **On the other hand, no measurement of treatment credibility was performed so we cannot ensure the success of participants blinding.** Finally, although this was a trial about chronic pain, the possible concomitant pharmacological analgesic treatment of patients was not considered. Nevertheless, the evaluation of medication is recognized as a complex aspect of patient outcomes and assessment of this domain is only recommended when it is the specific object of study [47].

## Conclusions

The application of KT in CLBP patients after first having identified skin/fascia mobilization as TEM during exploration, produced a significant reduction in pain and disability, at the post-treatment time point. **Reduction in both variables** continued to 6 moths only in the KT group, although **there were no differences between groups at any measurement time point**.

## Supporting information

S1 Checklist CONSORT 2010 checklist. (DOC)

S1 Protocol Trial protocol study_Spanish version. (DOCX)

S2 Protocol Trial protocol study_English version. (DOCX)
