# Supplementary material for: A novel (targeted) kinesio taping application on chronic low back pain: Randomized clinical trial
Source: PLoS One. 2021 May 13;16(5):e0250686. doi: 10.1371/journal.pone.0250686 (PMC8118519; doi:10.1371/journal.pone.0250686)
Supplement: S1 Protocol — (DOCX) [file pone.0250686.s002.docx]

**TRATAMIENTO CON VENDAJE NEUROMUSCULAR EN LUMBALGIA MECÁNICA CRÓNICA. ENSAYO CLÍNICO ALEATORIZADO.**

**RESUMEN**

**Objetivos.** Evaluar la eficacia del tratamiento con vendaje neuromuscular (VNM) en la intensidad del dolor, la discapacidad y la calidad de vida de pacientes con lumbalgia mecánica crónica (LMC). Los objetivos secundarios del estudio son evaluar el efecto del tratamiento con VNM en el patrón de actividad neuromuscular de la musculatura extensora del tronco, abdominales e isquiotibiales y analizar la influencia de las creencias de temor-evitación, de las ideas de catastrofización, y de la presencia de ansiedad-depresión en la eficacia del VNM en el tratamiento de estos pacientes.

**Metodología** Se trata de un ensayo clínico aleatorizado en 62 pacientes con LMC, reclutados en una consulta médica especializada de rehabilitación. Se ofertará participar en el estudio de forma consecutiva a todos aquellos pacientes en los que la movilización de piel/fascias en la exploración clínica se muestre como “factor modificador del efecto del tratamiento”. De forma aleatoria y con ocultación de la secuencia de aleatorización, serán asignados a dos grupos de intervención: grupo de VNM y grupo de vendaje placebo. La duración del tratamiento será de 4 semanas, realizándose el cambio del vendaje en 4 ocasiones, con una frecuencia semanal. En la valoración de resultados por una parte se tendrán en cuenta las alteraciones en los patrones de actividad muscular de los extensores del tronco, obtenidos mediante electromiografía con electrodos de superficie (SEMG). Por otra parte se obtendrán datos mediante entrevista clínica y el uso de cuestionarios validados: Escala Numérica de Dolor (END), Cuestionario Roland-Morris (RM), SF-36, Fear Avoidance Beliefs Questionnaire (FABQ), Hospital Anxiety and Depression subscale (HAD) y Escala de catastrofización del Dolor (CSQ). La valoración se realizará en 3 momentos evolutivos: preintervención, postintervención y a los 6 meses. Tanto los pacientes como los evaluadores permanecerán ciegos al tratamiento asignado.

**ANTECEDENTES Y ESTADO ACTUAL**

La lumbalgia mecánica inespecífica es en la actualidad uno de los principales problemas sanitarios de las sociedades desarrolladas, no sólo por su elevada prevalencia y por el elevado porcentaje de cronificación (1,2,3), sino sobre todo por el crecimiento exponencial de la discapacidad e invalidez secundarias a la misma que se ha producido en las últimas décadas, a pesar de haberse mantenido constante la prevalencia (4). Así, en España entre 1993 y 2004 se produjo un incremento de un 183% en el total de bajas laborales por este motivo y representa la principal causa de discapacidad en personas menores de 45 años (1,5).

En Medicina Física y Rehabilitación (MFyRHB) el tratamiento de los pacientes afectos de lumbalgia mecánica crónica (LMC) supone un verdadero reto. A pesar de ello, la falta de ensayos clínicos de calidad es un hecho. En la actualidad se puede afirmar que la terapia con ejercicios parece eficaz, aunque la evidencia está basada en estudios de baja calidad, y que la neuroestimulación eléctrica transcutánea es ineficaz. Sin embargo no se dispone de datos suficientes para establecer conclusiones firmes sobre el efecto clínico de otras técnicas empleadas habitualmente como educación en escuela de espalda, laserterapia de baja potencia, educación del paciente, masaje, tracción, calor/frío superficial, lumbostatos o manipulación (6,7).

En los últimos años hemos asistido a la irrupción en el mundo de la terapia física de una nueva técnica desarrollada por Kase, el método Kinesio Taping (8), conocido en España como vendaje neuromuscular (VNM). Inicialmente empleado en medicina deportiva, en la actualidad su ámbito de aplicación se ha ampliado de forma que puede ser utilizado en el tratamiento diario de numerosas patologías atendidas en MFyRHB (afecciones musculoesqueléticas, patología neurológica, linfedema y otras). Aunque el mecanismo de acción exacto por ahora es desconocido, se describen como efectos clínicos la mejoría de la microcirculación y del drenaje linfático, la normotonificación muscular, el soporte de las articulaciones, la activación de sistemas analgésicos endógenos y la influencia en órganos internos, con la gran ventaja de no limitar la movilidad articular (8).

En la actualidad, pese a su popularidad todavía existen pocas evidencias científicas que apoyen el empleo del VNM. Durante la última década ha aumentado progresivamente el número de publicaciones sobre su efecto de en áreas tan diversas como patología vascular, patología neurológica, pediatría y principalmente en patología musculoesquelética (9,10).

En relación a la eficacia del VNM en LMC recientemente se han publicado dos ECA. Paoloni y cols(11) evalúan el efecto en cuanto a dolor, discapacidad y fenómeno de flexión relajación lumbar (FFRL) en la valoración con electromiografía de superficie (EMGs) del VNM en LMC, comparándolo con ejercicios y con combinación de ejercicios-VNM. El VNM se muestra igual de eficaz que la terapia con ejercicios respecto a la reducción del dolor, no así en cuanto a discapacidad. Por otra parte en un 28% de los pacientes, sin diferencias entre grupos, se recupera el FFRL. Castro Sánchez et al (12) comparan el efecto en cuanto a dolor, discapacidad, resistencia de la musculatura del tronco y kinesiofobia del VNM frente a placebo. Este ECA muestra una disminución del dolor estadísticamente significativa, aunque clínicamente no relevante (1 cm en la EVA) a favor del VNM, así como una mejoría significativa en la resistencia de la musculatura del tronco frente a placebo. Aunque ambos ECA no muestran resultados clínicamente relevantes, sí apuntan una tendencia positiva hacia la eficacia del VNM en LMC. Además, como limitaciones de los estudios son de destacar la muestra reducida de pacientes (39 pacientes), la ausencia de grupo placebo, la evaluación a corto plazo (1 mes) y el diseño como simple ciego en el primero y la corta duración de la aplicación del tratamiento (1 semana) y la evaluación de los resultados a corto plazo (1 mes) en el segundo.

Un elemento central en el debate actual sobre la mejor práctica en el manejo de la LM es la eficacia de tratamientos dirigidos frente a tratamientos genéricos. Muchos clínicos e investigadores opinan que adaptar el tratamiento a subgrupos de pacientes impacta positivamente en la evolución clínica. Una revisión sistemática sobre la eficacia de los tratamientos dirigidos a subgrupos de pacientes con LM proporciona una evidencia cautelosa que apoya este enfoque terapéutico, en base a los resultados de un estudio de alta calidad que muestra un tamaño del efecto clínico estadísticamente significativo a corto plazo con la cinesiterapia de Mc Kenzie (ejercicios basados en la dirección preferente). Se consideran “modificadores del efecto del tratamiento” los síntomas y signos que indican una respuesta más probable a un tratamiento específico (13). Según Kase, el objetivo en la aplicación del vendaje es que este replique la posición de las manos del terapeuta en la piel del paciente (8). En patología musculoesquelética, durante la exploración clínica podemos incluir la movilización manual de la piel/fascias del paciente en diferentes direcciones. Si dicha movilización modifica la sintomatología o semiología del paciente se puede considerar como un “modificador del efecto del tratamiento”. A pesar de ello en ninguno de los estudios referenciados (11,12) tienen en cuenta este hecho, utilizando pautas de tratamiento estandarizado para todos los pacientes con LMC. Es posible que el VNM se muestre más eficaz cuando es aplicado a subgrupos de pacientes con LMC con características clínicas detectables en la exploración.

Más allá de los estudios sobre los efectos del tratamiento con VNM, se desconoce los posibles mecanismos en los que estos efectos podrían basarse. Kenzo Kase hipotetizó que uno de ellos podría ser la normalización de la actividad muscular. En el caso concreto de la región lumbar, las valoraciones electromiográficas de la actividad neuromuscular de la musculatura extensora de la espalda han sido de hecho utilizadas con frecuencia como medida objetiva del déficit, que ayuda en el diagnóstico de pacientes con LM. La mayoría de trabajos publicados se han centrado en el estudio del fenómeno de FFRL. Al final de la flexión lumbar se produce un silencio mioeléctrico en los erectores espinales, pasando a ser soportada la carga por las estructuras pasivas (14). En pacientes con LM dicho silencio desaparece, probablemente debido a que la contracción de los erectores aumentaría la estabilidad del raquis, deficitaria por la lesión de las estructuras pasivas del mismo (15). El FFRL medido mediante SEMG parece distinguir entre sujetos con LM y controles con buena precisión (16). Así mismo se ha mostrado sensible a los cambios funcionales relevantes tras el tratamiento en pacientes con LMC (17). Si realmente el VNM trabaja a través de una normalización de la actividad muscular, a nivel de la región lumbar podría causar alteraciones en los patrones típicos de ausencia de relajación que muestran los pacientes con dolor, como parece apuntar el ensayo de Paoloni y cols. (11).

Teniendo en cuenta todo lo expuesto, hemos diseñado un estudio para valorar la eficacia a medio plazo del tratamiento de LMC con VNM, aplicado durante 4 semanas a pacientes en los que la movilización de piel/fascias se muestra como un signo “modificador del efecto del tratamiento”.

**HIPÓTESIS**

- El tratamiento con VNM de pacientes afectos de LM crónica, en cuya exploración la movilización de piel/fascias se muestre como “factor modificador del efecto del tratamiento”, producirá una mejoría clínicamente relevante en relación al dolor, discapacidad y calidad de vida en comparación con vendaje placebo.
- El efecto del tratamiento con VNM se mantendrá a medio plazo (6 meses).

**OBJETIVOS**

- Objetivo principal: Evaluar la eficacia a corto y medio plazo del tratamiento con VNM en el dolor, la discapacidad y la calidad de vida en pacientes con LMC, mediante un estudio controlado y aleatorizado.
- Objetivos secundarios: 1) Evaluar el efecto del tratamiento con VNM en los patrones de actividad muscular de los extensores del tronco, abdominales e isquiotibiales obtenidos mediante electromiografía con electrodos de superficie. 2) Analizar la influencia de las creencias de temor-evitación, de las ideas de catastrofización, de la presencia de ansiedad-depresión y de las estrategias de afrontamiento del dolor en la eficacia del VNM en el tratamiento de pacientes con LM crónica.

**METODOLOGÍA**

**Diseño**

Ensayo clínico aleatorizado, doble ciego, con 2 brazos (VNM, placebo), 3 momentos de evaluación (pre-tratamiento, al finalizar el tratamiento y seguimiento a los 6 meses) y análisis por intención de tratar.

**Participantes**

Se invitará a participar en el estudio a pacientes diagnosticados de LM crónica reclutados de las consulta del Servicio de Rehabilitación de un área sanitaria de Valencia que atiende a una población de 320000 habitantes.

Deberán cumplir los siguientes criterios de inclusión: 1) Edad entre 18 y 65 años. 2) LMC de 6 meses mínimo de duración. 3) Mejoría del dolor con la movilización de piel/fascias en la exploración. 4) Firma de consentimiento informado.

Como criterios de exclusión se considerarán: 1) Haber tenido experiencia previa con tratamientos de VNM. 2) Presencia de componente de dolor neuropático (radiculopatía, estenosis de canal lumbar). 3) Dolor de origen específico (fractura vertebral, neoplasia en columna o estructuras nerviosas, espondiloartropatía , espondilodiscitis). 4) Cirugía previa de raquis. 5) Retraso mental, enfermedad mental grave, abuso o dependencia de sustancias, analfabetismo.

**Potencia y tamaño muestral**

El estudio se ha planeado para obtener una diferencia de 3 puntos en el cuestionario de Roland Morris ya que estos valores han sido reconocidos como clínicamente relevantes (18,19). Según los resultados de una muestra previa de pacientes con lumbalgia mecánica crónica obtenida en nuestro centro la discapacidad media medida por cuestionario RM tenía una SD de 3,2. Considerando un valor alfa de 0.05 y beta de 0.9 para comparación de medias de dos colas, el tamaño muestral requerido es de 24 pacientes por grupo para detectar cambios en discapacidad. Considerando unas pérdidas de 20% la muestra del estudio se establece en 62 pacientes (20).

**Diagrama de flujo**

Atendiendo a las directrices CONSORT (21), se dibujará un diagrama de flujo que describa la situación durante el curso del ensayo de todos los pacientes invitados, incluyendo el número de aquellos que rechazan participar antes de la aleatorización, la asignación a cada brazo de tratamiento, las pérdidas durante el seguimiento y los posibles entrecruzamientos.

**Intervención**

Los pacientes que accedan a participar en el estudio serán asignados aleatoriamente a uno de los dos grupos de intervención. La asignación la realizará una lista generada por ordenador (programa SPSS 19), balanceando a los participantes entre los grupos según el grado de discapacidad basal en la entrada del estudio, y siendo supervisada la aleatorización por un estadístico ajeno al equipo investigador. Se mantendrá la ocultación de la secuencia de aleatorización.

El grupo VNM recibirá el tratamiento con VNM de 5 cm de ancho, aplicado en función de la exploración clínica, en base a la siguiente pauta: Si el dolor lumbar en flexión o extensión mejora a) al movilizar la piel/fascias en sentido longitudinal a la musculatura paravertebral, se aplicará la técnica muscular, con la base colocada en el sentido del deslizamiento, sin tensión y en posición de máxima flexión lumbar posible, b) al movilizar la piel/fascias en aproximación hacia un punto, se aplicará la técnica de espacio (o ligamento), con tensión aproximada de un 75% y en posición de máxima flexión lumbar posible, c) al movilizar la piel/fascias en sentido transversal a la musculatura paravertebral, se aplicará la técnica de fascia, en posición de máxima flexión lumbar posible (pueden combinarse varias técnicas, en función de la exploración).

En el grupo placebo se aplicará la misma venda pero en posición corporal neutra, sin tensión y aplicada transversalmente en un nivel vertebral no doloroso a la palpación (12).

La duración del tratamiento será de 4 semanas, realizándose el cambio del vendaje en 4 ocasiones, con una frecuencia semanal.

**Medidas de resultados**:

En el ensayo clínico se tendrán en cuenta las siguientes variables:

- Datos sociodemográficos: edad, sexo, profesión (clasificación según CNAE), sedentarismo (sedentario, sedentario con deambulación, esfuerzo físico ligero o intenso), actividad deportiva (no deporte, deporte de bajo impacto, deporte de alto impacto; frecuencia semanal).
- Variables clínicas: peso y altura (IMC), tiempo de evolución del proceso (en meses), tratamiento farmacológico previo (tipo, dosis, duración), tratamiento físico previo (tipo, duración), exploraciones complementarias (Rx, TAC, RM)
- Valoración de resultados:

En la actualidad existe consenso en cuanto a las variables básicas a considerar en la evaluación de resultados en el tratamiento del dolor lumbar: dolor, limitación funcional específica por dolor lumbar, calidad de vida, discapacidad laboral y satisfacción (22,23,24). En base a estos consensos, en la valoración de resultados tendremos en cuenta las siguientes variables:

- Intensidad de dolor: Escala Numérica de Dolor (END), preferible a la escala visual analógica por ser menos abstracta y más fácil de entender (24).
- Estudio de la SEMG de erectores espinales lumbares, abdominales oblicuos/transversos, e isquiotibiales. El patrón de activación muscular se estudiará de manera descriptiva, comprobando la presencia o no de relajación en flexión máxima. Los patrones pre y post-tratamiento se compararán de nuevo comprobando la existencia o no de relajación, y mediante promedios y ratios de actividad electromiográfica.
- Limitaciones de la actividad: versión validada al español del Cuestionario Roland-Morris (RM), útil en situaciones de discapacidad leve y moderada, más propia de la población habitualmente atendida en las consultas de MFyRHB (25,26,27,28).
- Calidad de vida: versión validada al español del cuestionario SF-36 (29).
- Situación laboral: en activo en su trabajo habitual, en activo en un puesto laboral restringido, Incapacidad Temporal, Invalidez Permanente , en paro por sus problemas de salud, en paro por otros motivos, estudiante, ama de casa, jubilado (22).
- Creencias de temor-evitación: versión validada al español del cuestionario Fear Avoidance Beliefs Questionnaire (FABQ) (30,31) que mide creencias y actitudes respecto al potencial efecto del trabajo y de la actividad física sobre el dolor.
- Ansiedad y depresión: versión validada al español del cuestionario Hospital Anxiety and Depression subscale (HAD) (32,33,34) con utilidad demostrada en el screening de la posible presencia de estados de ansiedad y depresión.
- Ideas de Catastrofización ante el dolor: versión validada al español de la Pain Catastrophizing Scale (PCS), que comprende 3 dimensiones: rumiación, magnificación y desesperanza ante el dolor (35,36).
- Comorbilidades: Índice de Comorbilidad de Charlston (37).
- Cointervenciones: Se registrarán tratamientos paralelos que pudieran recibir los participantes (otros tratamientos fisioterápicos, farmacológicos, infiltraciones discales, facetarias o epidurales, TENS, electroterapia o cirugía).
- Efectos adversos: se registrarán los efectos adversos que se produzcan durante el tratamiento.

**Recogida y Análisis de datos**

Los datos sociodemográficos y las variables clínicas se recogerán mediante entrevista clínica y por acceso a la historia electrónica del paciente. Los cuestionarios así como la EMGs se obtendrán antes del tratamiento, al finalizar el tratamiento y a los 6 meses. Tanto los participantes como los evaluadores de los resultados serán desconocedores de la asignación a cada uno de los brazos de tratamiento. Los resultados primarios se obtendrán por análisis por intención de tratar.

Se obtendrán los estadísticos descriptivos expresando las variables continuas en media (desviación estándar) o mediana (cuartiles) según su normalidad o no, las variables categóricas en número (porcentaje) y expresando en todos los casos el intervalo de confianza del 95%. La distribución normal se comprobará por el test de Kolmogorov para determinar el empleo de pruebas paramétricas o no paramétricas. Para comparación de variables continuas entre grupos se empleará las pruebas de t de Student o la U de Mann-Whitney  y para variables categóricas el test de Chi cuadrado o  de Fisher según el tipo de distribución. Los cambios intragrupo en las variables continuas al finalizar el tratamiento y a los 6 meses se analizarán con la prueba de t de Student para muestras apareadas. Para evaluar si hay diferencias entre los dos grupos en el dolor, la discapacidad y en las diferentes variables se realizarán análisis de covarianza (ANCOVA) uno para cada variable dependiente  En todas las pruebas, la variable independiente será el grupo mientras que la variable covariada será el nivel basal de cada variable dependiente. Será necesario tener niveles comparables en ambos grupos para interpretar correctamente los resultados de ANCOVA. Por tanto, se realizarán varias pruebas de t de Student, una para cada variable dependiente considerando a cada grupo como variable independiente en cada caso. Para evaluar si los cambios en los 2 grupos se producen de manera diferente, se realizarán varios ANOVA, un análisis para cada variable dependiente. En todos los casos, la variable independiente será el grupo y el tiempo en que se recogen los datos. En este análisis se estudia solo la posible presencia de interacciones. Además, se evaluarán las correlaciones entre las variables de estudio mediante r de Pearson o Rho de Spearmann según normalidad. También se aplicarán dos modelos de Regresión lineal múltiple con dolor y discapacidad como variables dependientes y en ambos modelos edad, sexo, dolor, catastrofizacion, temor-evitación, baja laboral, tiempo de evolución y ansiedad-depresióncomo variables independientes.

**Privacidad y protección de datos**

Se utilizarán contraseñas personales y encriptación de datos del AES (Advanced Encryption Standard). El proyecto se ajustará a las directrices existentes en España y UE para la protección de los pacientes en los ensayos clínicos respecto a recogida, almacenamiento y custodia de datos personales.

**Aspectos éticos**

Los investigadores se adhieren a la Declaración de Helsinki sobre investigación médica. A todos los pacientes elegibles se les dará información oral y escrita (consentimiento informado) sobre el estudio y las dos modalidades de tratamiento. Específicamente se les informará que pueden abandonar el estudio en cualquier momento sin necesidad de dar explicación y que esta decisión no afectará la continuación de su tratamiento ordinario.

**Limitaciones del estudio**

Un criterio de exclusión en la selección de los pacientes es que hayan sido tratados previamente con VNM para poder cegarlos adecuadamente. No obstante, el hecho de que en el grupo placebo el vendaje se coloque en niveles segmentarios no dolorosos puede hacerles sospechar de su asignación, rompiéndose el carácter ciego del estudio, aunque se mantendrá oculta la secuencia de aleatorización.

El contexto de la recesión económica actual puede hacer que los efectos de la LMC sobre las bajas laborales estén minimizados en la muestra inicial y que el efecto beneficioso de las intervenciones sobre el retorno al trabajo esté magnificado.

**Aplicaciones/Relevancia del tema**

La LM es un proceso muy frecuente con una gran repercusión personal, sobre el gasto sanitario y sobre la sociedad, siendo la primera causa de baja laboral en menores de 45 años. En la actualidad no existe un tratamiento eficaz para la LMC, siendo necesario encontrar nuevas estrategias de tratamiento que mejoren los resultados. Si el tratamiento diseñado en este proyecto es efectivo, podría ser aplicado en la práctica clínica ordinaria de nuestra área sanitaria y ser diseminado por el SNS para mejorar el tratamiento de los pacientes con DL crónico.

Por otra parte, la amplia difusión de la aplicación del VNM en el área de la terapia física obliga a la realización de ensayos clínicos de calidad que permitan justificar de forma objetiva su empleo en diferentes procesos, entre ellos la LM. En la actualidad la mayoría de los tratamientos de rehabilitación para el dolor lumbar se están aplicando sin evidencias sólidas que justifiquen su empleo (38).

**BIBLIOGRAFÍA**

1. Deyo RA, Weinstein JN. Low back pain. N Engl J Med 2001; 344 (5): 363-70.
2. Von Korff M, Deyo RA, Cherkin D, Barlow W. Back pain in primary care: outcomes at one year. Spine 1993; 18: 855-62.
3. Von Korff M, Saunders K. The course of back pain in primary care. Spine 1996: 21: 2833-39.
4. Waddell G. The back pain revolution. Glasgow: Churchill & Livingstone, 2004.)
5. Salvans MM, González-Viejo MA. Incapacidad laboral por dolor lumbar en España de 2000 a 2004. Med Clin (Barc)2008; 131 (8): 319.
6. Van Middelkoop M, Rubinstein SM, Kuijpers T, Verhagen AP, Ostelo R, Koes BW, van Tulder MW. A systematic review on the effectiveness of physical and rehabilitation interventions for chronic non-specific low back pain. Eur Spine J (2011) 20:19–39.
7. Rubinstein SM, van Middelkoop M, Kuijpers T, Ostelo R, Verhagen AP, de Boer MR, Koes BW, van Tulder MW. A systematic review on the effectiveness of complementary and alternative medicine for chronic non-specific low-back painEur Spine J (2010) 19:1213–28.
8. Kase, K., Wallis, J. & Kase, T. 2003, Clinical therapeutic applications of the Kinesio taping method. Ken Ikai Co Ltd, Tokyo.
9. Espejo L,Apolo MD. Revisión bibliográfica de la efectividad del kinesiotaping. Rehabilitación (Madr); 2011: 148-58.
10. Basset K, Lingman SA, Ellis RF. The use and treatment efficacy of kinaesthetic taping for musculoskeletal conditions: a sistemattic review. NZ Journal of Phisiotherapy 2010; 38(2): 56-62.
11. Paoloni M, Bernetti A, Fratocchi G, Mangone M,Parrinello L, Del Pilar Cooper M, Sesto L, Disante L, Santilli V. Kinesio taping applied to lumbar muscles influences clinical and electromyografic characteristics in chronic low back pain patients. Eur J, Phys Rehabil Med 2011; 47: 237-44.
12. Castro-Sánchez AM, Lara-Palomo CL, Matarán-PeñarrochaGA, Fernández-Sánchez M, Sánchez-Labraca N, Arroyo-Morales M. Kinesio Taping reduces disability and pain slightly in chronic non-specific low back pain: a randomised trialJournal of Physiotherapy 2012; 58: 89-95.
13. Kent P, Mjøsund HL, Petersen DHD. Does targeting manual therapy and/or exercise improve patient outcomes in nonspecific low back pain? A systematic review. BMC Medicine 2010, 8:22.http://www.biomedcentral.com/1741-7015/8/22.
14. Foyd WF, Silver PHS. The function of erectors spinae muscles in certain movements and postures in man. J Physiol. 1995; 129: 184-203.
15. Colloca CJ, Hinrichs RN. The biomechanical and clinical significance of the lumbar erector spinae flexion-relaxation phenomenon: a review of literature. J Man Phys Ther 2005; 28 (8): 623-31.
16. Geisser ME, Ranavaya M, Haig AJ, Roth RS, Zucker R, Ambroz C, Caruso M. A meta-anlytic review of surface electromyography among persons with low back pain and normal, healthy controls. He Journal of pain 2005; 6(1): 711-26.
17. Mayer TG, Neblett R, Brede E, Gatchel RJ. The Quantified Lumbar Flexion-Relaxation Phenomenon Is a Useful Measurement of Improvement in a Functional Restoration Program. Spine 2009; 34 (22): 2458–65.)
18. Ostelo RW, Deyo RA, Stratford P, Waddell G, Croft P, Von Korff M, Bouter LM, de Vet HC. Interpreting change scores for pain and functional status in low back pain: towards international consensus regarding minimal important change. Spine 2008;33:90-4.
19. Deyo RA, Battie M, Beurskens AJ, Bombardier C, Croft P, Koes B, Malmivaara A, Roland M, Von Korff M, Waddell G. Outcome measures for low back pain research. A proposal for standardized use. Spine 1998;23:2003-13
20. Uitenbroek, Daan G, Binomial. SISA. 1997. http://www.quantitativeskills.com/sisa/distributions/binomial.htm. (9 April. 2012)
21. Schulz et al. CONSORT 2010 Statement: updated guidelines for reporting parallel group randomised trials. Trials 2010, 11:32 <http://www.trialsjournal.com/content/11/1/32>
22. Bombardier C. Outcomes assessments in the evaluation of treatment of spinal disorders. Summary and general recommendations. Spine 2000; 25(24): 3100-3.
23. Kovacs FM, Abraira V, Zamora J, Gil del Real MT, Llobera J, Fernández C and the Kovacs Atención Primaria Group. Correlation between pain, disability and quality of live in patients with common low back pain. Spine 2004; 29 (2): 206-10.
24. Dworkin RH, Turk DC, Farrar JT, Haythornthwaite JA, Jensen MP, Katz NP, Kerns RD, Stucki G, Allen RR, Bellamy N, Carr DB, Chandler J, Cowan P, Dionne R, Galer BS, Hertz S, Jadad AR, Kramer LD, Manning DC, Martin S, McCormick CG, McDermott MP, McGrath P, Quessy S, Rappaport BA, Robbins W, Robinson JP, Rothman M, Royal MA, Simon L, Stauffer JW, Stein W, Tollett J, Wernicke J, Witter J. Core outcome measures for chronic pain clinical trials: IMMPACT recommendations. Pain, 2005;113:9-19)
25. Roland , Fairbank J. The Roland-Morris disability Questionnaire and the Oswestry Disability Questionnaire. Spine 2000; 25 (24): 3115-24.
26. Kopec JA. Measuring functional outcomes in persons with back pain. A review of back-specific questionnaires. Spine 2000; 25 (24): 3110-4.
27. Kovacs FM, Llobera J, Gil del real MT, Abraira V, Gestoso M, Fernández C and the Kovcas-Atención primaria Group. Validation of the spanish versión of the Roland-Morris Questionnaire. Spine 2002; 27 (5): 538-42.
28. Grotle M, Brox JI, Vollestad NK. Functional status and disability questionnaires: what do they assess?. Spine 2005; 30 (1): 130-40.
29. Alonso J, Prieto L, Antó JM. La versión española del SF-36 Health Survey (Cuestionario de Salud SF-36): un instrumento para la medida de los resultados clínicos. Med Clin (Barc) 1995; 104: 771-6.
30. Waddell G, Newton M, Henderson I, Somerville D, Main CJ Fear-avoidance beliefs questionnaire and the role of fear avoidance beliefs in chronic low back pain and disability. Pain 1993; 52:157–68.
31. Kovacs FM, MD, Muriel A, Medina JM, Abraira V, PhD, Castillo Sánchez MD, Olabe Jaúregui J and the Spanish Back Pain Research Network. Psychometric Characteristics of the Spanish Version of the FAB Questionnaire. Spine 2006;31:104–10.
32. Zigmond AS, Snaith RP. The Hospital Anxiety and Depression Scale. Acta Psychiatr Scand 1983;67:361–70.
33. Bjelland I, Dahl AA, Haug TT, Neckelmann D. The validity of the Hospital Anxiety and Depression Scale An updated literature review. Journal of Psychosomatic Research 2002; 52: 69– 77
34. Herrero MJ, Blanch J, Peri JM, De Pablo J, Pintor L, Bulbena A. A validation study of the hospital anxiety and depression scale (HADS) in a Spanish population. General Hospital Psychiatry 2003; 25: 277–83.
35. Sullivan MJL, Stanish W, Waite H, Sullivan M, Tripp DA. Catastrophizing, pain, and disability following soft tissue injuries. Pain. 1998;77:253-60.
36. García Campayo J, Rodero B, Alda M, Sobradiel N, Montero J, Moreno S. Validación de la versión española de la escala de la catastrofización ante el dolor (Pain Catastrophizing Scale) en la fibromialgia. Med Clin (Barc). 2008;131(13):487-92.
37. Charlson ME, Pompei P, Ales KL, MacKenzie CR. A new method of classifying prognostic comorbidity in longitudinal studies: development and validation. J Chronic Dis. 1987;40(5):373-83.
38. Serrano-Aguilar P, Kovacs FM, Cabrera-Hernández JM, Ramos-Goñi JM, García-Pérez L. Avoidable costs of physical treatments for chronic back, neck and shoulder pain within the Spanish National Health Service: a cross-sectional study. BMC Musculoskelet Disord. 2011 Dec 21;12:287.
