# Supplementary material for: A novel (targeted) kinesio taping application on chronic low back pain: Randomized clinical trial
Source: PLoS One. 2021 May 13;16(5):e0250686. doi: 10.1371/journal.pone.0250686 (PMC8118519; doi:10.1371/journal.pone.0250686)
Supplement: S2 Protocol — (DOCX) [file pone.0250686.s003.docx]

**TREATMENT WITH NEUROMUSCULAR BANDAGE IN CHRONIC MECHANICAL LOW BACK PAIN. RANDOMIZED CLINICAL TRIAL.**

**ABSTRACT**

**Objectives.** To assess the efficacy of neuromuscular bandage (NMV) treatment on pain intensity, disability and quality of life in patients with chronic mechanical low back pain (CMLBP). The secondary objectives of the study are to evaluate the effect of treatment with VNM on the neuromuscular activity pattern of the extensor muscles of the trunk, abdominals and hamstrings and to analyze the influence of fear-avoidance beliefs, ideas of catastrophization, and presence of anxiety-depression in the efficacy of NMV in the treatment of these patients.

**Methods.** This is a randomized clinical trial in 62 patients with CMLBP, recruited from a specialized rehabilitation medical practice. Participation in the study will be offered consecutively to all those patients in whom the mobilization of skin / fascia in the clinical examination is shown as a "modifying factor of the effect of the treatment". They will be assigned randomly and with concealment of the randomization sequence to two intervention groups: the VNM group and the placebo bandage group. The duration of treatment will be 4 weeks, changing the bandage 4 times, weekly. In evaluating the results, on the one hand, alterations in the muscle activity patterns of the trunk extensors, obtained by surface electrode electromyography (SEMG), will be taken into account. On the other hand, data will be obtained by clinical interview and the use of validated questionnaires: Numerical Pain Scale (NPS), Roland-Morris Questionnaire (RM), SF-36, Fear Avoidance Beliefs Questionnaire (FABQ), Hospital Anxiety and Depression subscale (HAD) and Pain Catastrophization Scale (PCS). The assessment will be carried out in 3 evolutionary moments: pre-intervention, post-intervention and at 6 months. Both patients and evaluators will remain blind to the assigned treatment.

**BACKGROUND**

Nonspecific mechanical low back pain is currently one of the main health problems in developed societies, not only because of its high prevalence and the high percentage of chronicity (1,2,3), but above all because of the exponential growth of disability and disability secondary to the same that has occurred in recent decades, despite the prevalence having remained constant (4). Thus, in Spain between 1993 and 2004 there was an increase of 183% in total sick leave for this reason and it represents the main cause of disability in people under 45 years of age (1,5).

In Physical Medicine and Rehabilitation (MFyRHB) the treatment of patients with CMLBP is a real challenge. Despite this, the lack of quality clinical trials is a fact. At present, it can be stated that exercise therapy appears effective, although the evidence is based on low-quality studies, and that transcutaneous electrical neurostimulation is ineffective. However, insufficient data are available to draw firm conclusions about the clinical effect of other commonly used techniques such as back school education, low-power laser therapy, patient education, massage, traction, superficial heat / cold, lumbostats or manipulation ( 6.7).

In recent years we have witnessed the emergence in the world of physical therapy of a new technique developed by Kase, the Kinesio Taping method (8), known in Spain as neuromuscular bandage (VNM). Initially used in sports medicine, its scope of application has now been expanded so that it can be used in the daily treatment of numerous pathologies treated in MFyRHB (musculoskeletal conditions, neurological pathology, lymphedema and others). Although the exact mechanism of action is unknown for now, the clinical effects are the improvement of microcirculation and lymphatic drainage, muscle normotonification, joint support, activation of endogenous analgesic systems and influence on internal organs, with the great advantage of not limiting joint mobility (8).

Currently, despite its popularity, there is still little scientific evidence to support the use of VNM. During the last decade, the number of publications on its effect in areas as diverse as vascular pathology, neurological pathology, pediatrics and mainly in musculoskeletal pathology has progressively increased (9,10).

In relation to the efficacy of NMV in CMLBP, two RCTs have recently been published. Paoloni et al (11) evaluated the effect in terms of pain, disability and the phenomenon of lumbar flexion relaxation (FFRL) in the assessment with surface electromyography (EMGs) of NMV in CMLBP, comparing it with exercises and with a combination of exercises-NMV. NMV is just as effective as exercise therapy in reducing pain, but not in disability. On the other hand, in 28% of the patients, without differences between groups, the FFRL is recovered. Castro Sánchez et al (12) compared the effect in terms of pain, disability, resistance of the trunk musculature and kinesiophobia of NMV versus placebo. This RCT shows a statistically significant decrease in pain, although not clinically relevant (1 cm in the VAS) in favor of the NMV, as well as a significant improvement in the resistance of the trunk musculature compared to placebo. Although both RCTs do not show clinically relevant results, they do point to a positive trend towards the efficacy of NMV in CMLBP. Furthermore, limitations of the studies include the small sample of patients (39 patients), the absence of a placebo group, the short-term evaluation (1 month), and the single-blind design in the first and the short duration of the study. application of treatment (1 week) and evaluation of short-term results (1 month) in the second.

A central element in the current debate on best practice in the management of SCI is the efficacy of targeted versus generic treatments. Many clinicians and researchers believe that tailoring treatment to subgroups of patients positively impacts clinical outcome. A systematic review on the efficacy of treatments targeting subgroups of SCI patients provides cautious evidence supporting this therapeutic approach, based on the results of a high-quality study showing a statistically significant short-term clinical effect size with Mc Kenzie's kinesitherapy (exercises based on preferred direction). Symptoms and signs indicating a more likely response to a specific treatment are considered “modifiers of treatment effect” (13). According to Kase, the objective in applying the bandage is that it replicates the position of the therapist's hands on the patient's skin (8). In musculoskeletal pathology, during the clinical examination we can include manual mobilization of the patient's skin / fascia in different directions. If said mobilization modifies the symptoms or semiology of the patient, it can be considered as a “modifier of the effect of the treatment”. Despite this, none of the referenced studies (11,12) take this fact into account, using standardized treatment guidelines for all patients with CMLBP. It is possible that NMV is more effective when applied to subgroups of patients with CMLBP with detectable clinical features on examination.

Beyond studies on the effects of NMV treatment, the possible mechanisms on which these effects could be based are unknown. Kenzo Kase hypothesized that one of them could be the normalization of muscle activity. In the specific case of the lumbar region, electromyographic assessments of the neuromuscular activity of the extensor muscles of the back have in fact been frequently used as an objective measure of deficit, which helps in the diagnosis of patients with SCI. Most of the published works have focused on the study of the FFRL phenomenon. At the end of lumbar flexion, a myoelectric silence occurs in the spinal erectors, the load being supported by the passive structures (14). In patients with SCI, this silence disappears, probably because the contraction of the erectors would increase the stability of the spine, deficient due to the damage to its passive structures (15). The FFRL measured by SEMG seems to distinguish between subjects with SCI and controls with good precision (16). Likewise, it has been shown to be sensitive to relevant functional changes after treatment in patients with CMLBP (17). If the NMV really works through a normalization of muscle activity, at the level of the lumbar region it could cause alterations in the typical patterns of absence of relaxation shown by patients with pain, as the trial by Paoloni et al. Seems to point out. (11).

Taking into account all of the above, we have designed a study to assess the medium-term efficacy of CMLBP treatment with NMV, applied for 4 weeks to patients in whom mobilization of skin / fasciae is shown as a "modifying sign of the effect of treatment".

**HYPOTHESIS**

- Treatment with VNM in patients with CMLBP, in whose examination the mobilization of skin / fasciae is shown as a "modifying factor of the effect of the treatment", will produce a clinically relevant improvement in relation to pain, disability and quality of life in comparison with placebo bandage.

- The effect of treatment with VNM will be maintained in the medium term (6 months).

**OBJECTIVES**

- Main objective: To evaluate the short and medium term efficacy of NMV treatment in pain, disability and quality of life in patients with CMLBP, through a controlled and randomized study.

- Secondary objectives: 1) To evaluate the effect of NMV treatment on the muscle activity patterns of the trunk, abdominal and hamstring extensors obtained by electromyography with surface electrodes. 2) To analyze the influence of fear-avoidance beliefs, of catastrophizing ideas, of the presence of anxiety-depression, and of pain-coping strategies on the effectiveness of the VNM in the treatment of patients with CMLBP.

**METHODS**

**Design**

Randomized, double-blind clinical trial, with 2 arms (VNM, Vplacebo), 3 assessing times (pre-treatment, at the end of treatment and follow-up at 6 months) and intention-to-treat analysis.

**Participants**

Patients diagnosed with CMLBP recruited from the consultations of the Rehabilitation Service of a health area in Valencia that serve a population of 320,000 inhabitants will be invited to participate in the study.

They must meet the following inclusion criteria: 1) Age between 18 and 65 years. 2) LMC of 6 months minimum duration. 3) Amelioration of pain with mobilization of skin / fascia on examination. 4) Signature of informed consent.

The following will be considered as exclusion criteria: 1) Having had previous experience with NMV treatments. 2) Presence of neuropathic pain component (radiculopathy, lumbar canal stenosis). 3) Pain of specific origin (vertebral fracture, neoplasia in the spine or nervous structures, spondyloarthropathy, spondylodiscitis). 4) Previous spine surgery. 5) Mental retardation, severe mental illness, substance abuse or dependence, illiteracy.

**Power and sample size**

The study has been planned to obtain a difference of 3 points in the Roland Morris questionnaire since these values have been recognized as clinically relevant (18,19). According to the results of a previous sample of patients with CMLBP obtained in our center, the mean disability measured by the RM questionnaire had a SD of 3.2. Considering an alpha value of 0.05 and beta of 0.9 for comparison of two-tailed means, the required sample size is 24 patients per group to detect changes in disability. Considering losses of 20%, the study sample is established in 62 patients (20).

**Participants flow chart**

Following the CONSORT guidelines (21), a flow chart will be drawn describing the situation during the course of the trial of all invited patients, including the number of those who refused to participate before randomization, the assignment to each treatment arm, losses to follow-up and possible crossovers.

**Intervention**

Patients who agree to participate in the study will be randomly assigned to one of the two intervention groups. The assignment will be made by a computer-generated list (SPSS 19 program), balancing the participants between the groups according to the degree of baseline disability at study entry, and the randomization being supervised by a statistician outside the research team. The randomization sequence will be concealed.

The NMV group will receive treatment with 5 cm wide NMV, applied according to the clinical examination, based on the following guideline: If the lumbar pain in flexion or extension improves a) when mobilizing the skin / fascia longitudinally to the paravertebral musculature, the muscular technique will be applied, with the base positioned in the sliding direction, without tension and in a position of maximum lumbar flexion possible, b) when mobilizing the skin / fasciae in approximation towards a point, the technique of space (or ligament), with approximately 75% tension and in a position of maximum possible lumbar flexion, c) when mobilizing the skin / fasciae transversely to the paravertebral musculature, the fascia technique will be applied, in a position of maximum flexion lumbar possible (several techniques can be combined, depending on the examination).

In the placebo group, the same bandage will be applied but in a neutral body position, without tension and applied transversely at a vertebral level that is not painful on palpation (12).

The duration of treatment will be 4 weeks, changing the bandage 4 times, weekly.

**Outcome measures**:

The following variables will be taken into account in the clinical trial:

- Sociodemographic data: age, sex, profession (classification according to CNAE), sedentary lifestyle (sedentary, sedentary with walking, light or intense physical effort), sporting activity (no sport, low-impact sport, high-impact sport; weekly frequency).

- Clinical variables: weight and height (BMI), process evolution time (in months), previous pharmacological treatment (type, dose, duration), previous physical treatment (type, duration), complementary examinations (Rx, CT, MRI)

- Evaluation of results:

Currently, there is consensus regarding the basic variables to be considered in the evaluation of results in the treatment of low back pain: pain, specific functional limitation due to low back pain, quality of life, work disability and satisfaction (22,23,24). Based on these consensuses, in the evaluation of results we will take into account the following variables:

- Pain intensity: Numerical Pain Scale (NPS), preferable to the visual analog scale because it is less abstract and easier to understand (24).
- Study of SEMG of lumbar spinal erectors, oblique / transverse abdominals, and hamstrings. The muscle activation pattern will be studied descriptively, checking the presence or absence of relaxation in maximum flexion. The pre and post-treatment patterns will be compared again, checking the existence or not of relaxation, and using averages and ratios of electromyographic activity.

• Limitations of the activity: validated version in Spanish of the Roland-Morris Questionnaire (RMQ), useful in situations of mild and moderate disability, more typical of the population usually attended in the MFyRHB consultations (25,26,27,28).

• Quality of life: validated Spanish version of the SF-36 questionnaire (29).

• Work situation: active in their usual job, active in a restricted job, Temporary Disability, Permanent Disability, unemployed due to health problems, unemployed for other reasons, student, housewife, retired (22).

• Fear-avoidance beliefs: validated Spanish version of the Fear Avoidance Beliefs Questionnaire (FABQ) (30,31) that measures beliefs and attitudes regarding the potential effect of work and physical activity on pain.

• Anxiety and depression: validated Spanish version of the Hospital Anxiety and Depression subscale (HAD) questionnaire (32,33,34) with demonstrated utility in screening for the possible presence of anxiety and depression states.

• Ideas of Catastrophizing in the face of pain: validated Spanish version of the Pain Catastrophizing Scale (PCS), which comprises 3 dimensions: rumination, magnification and hopelessness in the face of pain (35,36).

• Comorbidities: Charlston Comorbidity Index (37).

• Co-interventions: Parallel treatments that the participants may receive (other physiotherapeutic, pharmacological treatments, disc, facet or epidural infiltrations, TENS, electrotherapy or surgery) will be recorded.

• Adverse effects: adverse effects that occur during treatment will be recorded.

**Data collection and analysis**

Sociodemographic data and clinical variables will be collected by clinical interview and by accessing the electronic history of the patient. The questionnaires as well as the EMGs will be obtained before the treatment, at the end of the treatment and at 6 months. Both the participants and the outcome assessors will be unaware of the allocation to each of the treatment arms. Primary outcomes will be obtained by intention-to-treat analysis.

Descriptive statistics will be obtained by expressing continuous variables as mean (standard deviation) or median (quartiles) according to their normality or not, categorical variables in number (percentage) and expressing in all cases the 95% confidence interval. The normal distribution will be verified by the Kolmogorov test to determine the use of parametric or non-parametric tests. For comparison of continuous variables between groups, the Student's t tests or the Mann-Whitney U tests will be used and for categorical variables the Chi-square or Fisher's test according to the type of distribution. Intragroup changes in continuous variables at the end of treatment and at 6 months will be analyzed with Student's t test for paired samples. To assess whether there are differences between the two groups in pain, disability and in the different variables, analysis of covariance (ANCOVA) will be performed, one for each dependent variable. In all the tests, the independent variable will be the group while the covariate variable will be the baseline level of each dependent variable. It will be necessary to have comparable levels in both groups to correctly interpret the ANCOVA results. Therefore, several Student's t tests will be performed, one for each dependent variable, considering each group as an independent variable in each case. To assess whether the changes in the 2 groups occur differently, several ANOVAs will be performed, one analysis for each dependent variable. In all cases, the independent variable will be the group and the time in which the data are collected. In this analysis, only the possible presence of interactions is studied. In addition, the correlations between the study variables will be evaluated using Pearson's r or Spearmann's Rho according to normality. Two models of multiple linear regression will also be applied with pain and disability as dependent variables and in both models age, sex, pain, catastrophization, fear-avoidance, sick leave, evolution time and anxiety-depression as independent variables.

**Privacy and data protection**

Personal passwords and AES (Advanced Encryption Standard) data encryption will be used. The project will comply with the existing guidelines in Spain and the EU for the protection of patients in clinical trials regarding the collection, storage and custody of personal data.

**Ethical aspects**

Researchers adhere to the Declaration of Helsinki on medical research. All eligible patients will be given oral and written information (informed consent) about the study and the two treatment modalities. Specifically, they will be informed that they can leave the study at any time without explanation and that this decision will not affect the continuation of their routine treatment.

**Study limitations**

An exclusion criterion in the selection of patients is that they have been previously treated with NMV in order to be able to adequately blind them. However, the fact that in the placebo group the bandage is placed at non-painful segmental levels may make them suspicious of its allocation, breaking the blind nature of the study, although the randomization sequence will be kept hidden.

The context of the current economic recession may mean that the effects of the CMLBP on sick leave are minimized in the initial sample and that the beneficial effect of the interventions on the return to work is magnified.

**Applications / Relevance of the study**

CMLBP is a very frequent process with a great personal impact, on health spending and on society, being the first cause of sick leave in those under 45 years of age.Currently, there is no effective treatment for CMLBP, and new treatment strategies have to be found to improve outcomes. If the treatment designed in this project is effective, it could be applied in the ordinary clinical practice of our health area and be disseminated by the SNS to improve the treatment of patients with CMLBP.

On the other hand, the wide dissemination of the application of VNM in the area of physical therapy requires the performance of quality clinical trials that allow objectively justifying its use in different processes, including CMLBP. At present, most of the rehabilitation treatments for low back pain are being applied without solid evidence to justify their use (38).

**REFERENCES**

1. Deyo RA, Weinstein JN. Low back pain. N Engl J Med 2001; 344 (5): 363-70.
2. Von Korff M, Deyo RA, Cherkin D, Barlow W. Back pain in primary care: outcomes at one year. Spine 1993; 18: 855-62.
3. Von Korff M, Saunders K. The course of back pain in primary care. Spine 1996: 21: 2833-39.
4. Waddell G. The back pain revolution. Glasgow: Churchill & Livingstone, 2004.)
5. Salvans MM, González-Viejo MA. Incapacidad laboral por dolor lumbar en España de 2000 a 2004. Med Clin (Barc)2008; 131 (8): 319.
6. Van Middelkoop M, Rubinstein SM, Kuijpers T, Verhagen AP, Ostelo R, Koes BW, van Tulder MW. A systematic review on the effectiveness of physical and rehabilitation interventions for chronic non-specific low back pain. Eur Spine J (2011) 20:19–39.
7. Rubinstein SM, van Middelkoop M, Kuijpers T, Ostelo R, Verhagen AP, de Boer MR, Koes BW, van Tulder MW. A systematic review on the effectiveness of complementary and alternative medicine for chronic non-specific low-back painEur Spine J (2010) 19:1213–28.
8. Kase, K., Wallis, J. & Kase, T. 2003, Clinical therapeutic applications of the Kinesio taping method. Ken Ikai Co Ltd, Tokyo.
9. Espejo L,Apolo MD. Revisión bibliográfica de la efectividad del kinesiotaping. Rehabilitación (Madr); 2011: 148-58.
10. Basset K, Lingman SA, Ellis RF. The use and treatment efficacy of kinaesthetic taping for musculoskeletal conditions: a sistemattic review. NZ Journal of Phisiotherapy 2010; 38(2): 56-62.
11. Paoloni M, Bernetti A, Fratocchi G, Mangone M,Parrinello L, Del Pilar Cooper M, Sesto L, Disante L, Santilli V. Kinesio taping applied to lumbar muscles influences clinical and electromyografic characteristics in Chronic mechanical low back pain patients. Eur J, Phys Rehabil Med 2011; 47: 237-44.
12. Castro-Sánchez AM, Lara-Palomo CL, Matarán-PeñarrochaGA, Fernández-Sánchez M, Sánchez-Labraca N, Arroyo-Morales M. Kinesio Taping reduces disability and pain slightly in chronic non-specific low back pain: a randomised trialJournal of Physiotherapy 2012; 58: 89-95.
13. Kent P, Mjøsund HL, Petersen DHD. Does targeting manual therapy and/or exercise improve patient outcomes in nonspecific low back pain? A systematic review. BMC Medicine 2010, 8:22.http://www.biomedcentral.com/1741-7015/8/22.
14. Foyd WF, Silver PHS. The function of erectors spinae muscles in certain movements and postures in man. J Physiol. 1995; 129: 184-203.
15. Colloca CJ, Hinrichs RN. The biomechanical and clinical significance of the lumbar erector spinae flexion-relaxation phenomenon: a review of literature. J Man Phys Ther 2005; 28 (8): 623-31.
16. Geisser ME, Ranavaya M, Haig AJ, Roth RS, Zucker R, Ambroz C, Caruso M. A meta-anlytic review of surface electromyography among persons with low back pain and normal, healthy controls. He Journal of pain 2005; 6(1): 711-26.
17. Mayer TG, Neblett R, Brede E, Gatchel RJ. The Quantified Lumbar Flexion-Relaxation Phenomenon Is a Useful Measurement of Improvement in a Functional Restoration Program. Spine 2009; 34 (22): 2458–65.)
18. Ostelo RW, Deyo RA, Stratford P, Waddell G, Croft P, Von Korff M, Bouter LM, de Vet HC. Interpreting change scores for pain and functional status in low back pain: towards international consensus regarding minimal important change. Spine 2008;33:90-4.
19. Deyo RA, Battie M, Beurskens AJ, Bombardier C, Croft P, Koes B, Malmivaara A, Roland M, Von Korff M, Waddell G. Outcome measures for low back pain research. A proposal for standardized use. Spine 1998;23:2003-13
20. Uitenbroek, Daan G, Binomial. SISA. 1997. http://www.quantitativeskills.com/sisa/distributions/binomial.htm. (9 April. 2012)
21. Schulz et al. CONSORT 2010 Statement: updated guidelines for reporting parallel group randomised trials. Trials 2010, 11:32 <http://www.trialsjournal.com/content/11/1/32>
22. Bombardier C. Outcomes assessments in the evaluation of treatment of spinal disorders. Summary and general recommendations. Spine 2000; 25(24): 3100-3.
23. Kovacs FM, Abraira V, Zamora J, Gil del Real MT, Llobera J, Fernández C and the Kovacs Atención Primaria Group. Correlation between pain, disability and quality of live in patients with common low back pain. Spine 2004; 29 (2): 206-10.
24. Dworkin RH, Turk DC, Farrar JT, Haythornthwaite JA, Jensen MP, Katz NP, Kerns RD, Stucki G, Allen RR, Bellamy N, Carr DB, Chandler J, Cowan P, Dionne R, Galer BS, Hertz S, Jadad AR, Kramer LD, Manning DC, Martin S, McCormick CG, McDermott MP, McGrath P, Quessy S, Rappaport BA, Robbins W, Robinson JP, Rothman M, Royal MA, Simon L, Stauffer JW, Stein W, Tollett J, Wernicke J, Witter J. Core outcome measures for chronic pain clinical trials: IMMPACT recommendations. Pain, 2005;113:9-19)
25. Roland , Fairbank J. The Roland-Morris disability Questionnaire and the Oswestry Disability Questionnaire. Spine 2000; 25 (24): 3115-24.
26. Kopec JA. Measuring functional outcomes in persons with back pain. A review of back-specific questionnaires. Spine 2000; 25 (24): 3110-4.
27. Kovacs FM, Llobera J, Gil del real MT, Abraira V, Gestoso M, Fernández C and the Kovcas-Atención primaria Group. Validation of the spanish versión of the Roland-Morris Questionnaire. Spine 2002; 27 (5): 538-42.
28. Grotle M, Brox JI, Vollestad NK. Functional status and disability questionnaires: what do they assess?. Spine 2005; 30 (1): 130-40.
29. Alonso J, Prieto L, Antó JM. La versión española del SF-36 Health Survey (Cuestionario de Salud SF-36): un instrumento para la medida de los resultados clínicos. Med Clin (Barc) 1995; 104: 771-6.
30. Waddell G, Newton M, Henderson I, Somerville D, Main CJ Fear-avoidance beliefs questionnaire and the role of fear avoidance beliefs in Chronic mechanical low back pain and disability. Pain 1993; 52:157–68.
31. Kovacs FM, MD, Muriel A, Medina JM, Abraira V, PhD, Castillo Sánchez MD, Olabe Jaúregui J and the Spanish Back Pain Research Network. Psychometric Characteristics of the Spanish Version of the FAB Questionnaire. Spine 2006;31:104–10.
32. Zigmond AS, Snaith RP. The Hospital Anxiety and Depression Scale. Acta Psychiatr Scand 1983;67:361–70.
33. Bjelland I, Dahl AA, Haug TT, Neckelmann D. The validity of the Hospital Anxiety and Depression Scale An updated literature review. Journal of Psychosomatic Research 2002; 52: 69– 77
34. Herrero MJ, Blanch J, Peri JM, De Pablo J, Pintor L, Bulbena A. A validation study of the hospital anxiety and depression scale (HADS) in a Spanish population. General Hospital Psychiatry 2003; 25: 277–83.
35. Sullivan MJL, Stanish W, Waite H, Sullivan M, Tripp DA. Catastrophizing, pain, and disability following soft tissue injuries. Pain. 1998;77:253-60.
36. García Campayo J, Rodero B, Alda M, Sobradiel N, Montero J, Moreno S. Validación de la versión española de la escala de la catastrofización ante el dolor (Pain Catastrophizing Scale) en la fibromialgia. Med Clin (Barc). 2008;131(13):487-92.
37. Charlson ME, Pompei P, Ales KL, MacKenzie CR. A new method of classifying prognostic comorbidity in longitudinal studies: development and validation. J Chronic Dis. 1987;40(5):373-83.
38. Serrano-Aguilar P, Kovacs FM, Cabrera-Hernández JM, Ramos-Goñi JM, García-Pérez L. Avoidable costs of physical treatments for chronic back, neck and shoulder pain within the Spanish National Health Service: a cross-sectional study. BMC Musculoskelet Disord. 2011 Dec 21;12:287.
